# On the use of deep learning for phase recovery

**DOI:** 10.1038/s41377-023-01340-x

**Published:** 2024-01-01

**Authors:** Kaiqiang Wang, Li Song, Chutian Wang, Zhenbo Ren, Guangyuan Zhao, Jiazhen Dou, Jianglei Di, George Barbastathis, Renjie Zhou, Jianlin Zhao, Edmund Y. Lam

**Affiliations:** 1 Department of Electrical and Electronic Engineering, The University of Hong Kong, Hong Kong SAR, China; 2 School of Physical Science and Technology, Northwestern Polytechnical University, Xi’an, China; 3 Department of Biomedical Engineering, The Chinese University of Hong Kong, Hong Kong SAR, China; 4 School of Information Engineering, Guangdong University of Technology, Guangzhou, China; 5 Department of Mechanical Engineering, Massachusetts Institute of Technology, Cambridge, MA USA

**Keywords:** Imaging and sensing, Optical metrology

## Abstract

Phase recovery (PR) refers to calculating the phase of the light field from its intensity measurements. As exemplified from quantitative phase imaging and coherent diffraction imaging to adaptive optics, PR is essential for reconstructing the refractive index distribution or topography of an object and correcting the aberration of an imaging system. In recent years, deep learning (DL), often implemented through deep neural networks, has provided unprecedented support for computational imaging, leading to more efficient solutions for various PR problems. In this review, we first briefly introduce conventional methods for PR. Then, we review how DL provides support for PR from the following three stages, namely, pre-processing, in-processing, and post-processing. We also review how DL is used in phase image processing. Finally, we summarize the work in DL for PR and provide an outlook on how to better use DL to improve the reliability and efficiency of PR. Furthermore, we present a live-updating resource (https://github.com/kqwang/phase-recovery) for readers to learn more about PR.

## Abstract (web summary)

Deep learning for phase recovery including “pre-processing”, “in-processing”, “post-processing”, and “phase processing”.

## Graphical abstract

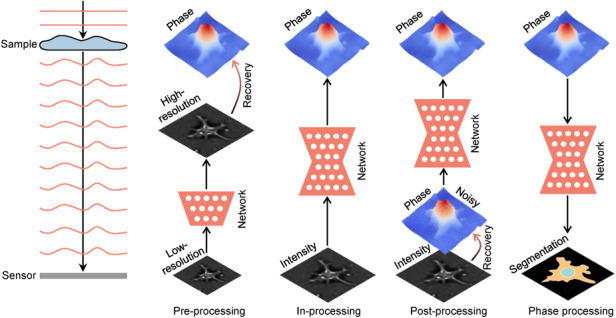

## Introduction

**Fig. 1:**
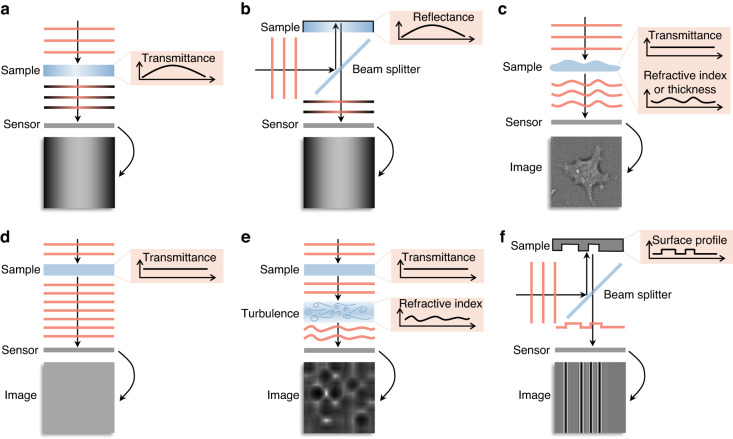
Light is transmitted through or reflected from different samples. **a** An absorptive sample with a nonuniform transmittance distribution. **b** A reflective sample with a nonuniform reflectance distribution. **c** A transparent (weakly-absorbing) sample with a nonuniform RI or thickness distribution. **d** A sample with a uniform transmittance distribution. **e** A sample with a uniform transmittance distribution placed before atmospheric turbulence with inhomogeneous RI distribution. **f** A reflective sample with a nonuniform surface height distribution

Light, as an electromagnetic wave, has two essential components: amplitude and phase^1^. Optical detectors, usually relying on photon-to-electron conversion (such as charge-coupled device sensors and the human eye), measure the intensity that is proportional to the square of the amplitude of the light field, which in turn relates to the transmittance or reflectance distribution of the sample (Fig. 1a, b). However, they cannot capture the phase of the light field because of their limited sampling frequency^2^.

Actually, in many application scenarios, the phase rather than the amplitude of the light field carries the primary information of the samples^3–6^. For quantitative structural determination of transparent and weakly scattering samples^3^ (Fig. 1c), the phase delay is proportional to the sample’s thickness or refractive index (RI) distribution, which is critically important for bioimaging because most living cells are transparent. For quantitative characterization of the aberrated wavefront^5^ (Fig. 1d, e), the phase aberration is caused by atmospheric turbulence with an inhomogeneous RI distribution in the light path, which is mainly used in adaptive aberration correction. Also, for quantitative measurement of the surface profile^6^ (Fig. 1f), the phase delay is proportional to the surface height of the sample, which is very useful in material inspection.

Since the phase delay across the wavefront is necessary for the above applications, but the optical detection devices can only perceive and record the amplitude of the light field, how can we recover the desired phase? Fortunately, as the light field propagates, the phase delay also causes changes in the amplitude distribution; therefore, we can record the amplitude of the propagated light field and then calculate the corresponding phase. This operation generally comes under different names according to the application domain; for example, it is quantitative phase imaging (QPI) in biomedicine^3^, phase retrieval in coherent diffraction imaging (CDI)^4^ which is the most commonly used term in X-ray optics and non-optical analogs such as electrons and other particles, and wavefront sensing in adaptive optics (AO)^5^ for astronomy and optical communications. Here, we collectively refer to the way of *calculating the phase of a light field from its intensity measurements* as phase recovery (PR).

**Fig. 2:**
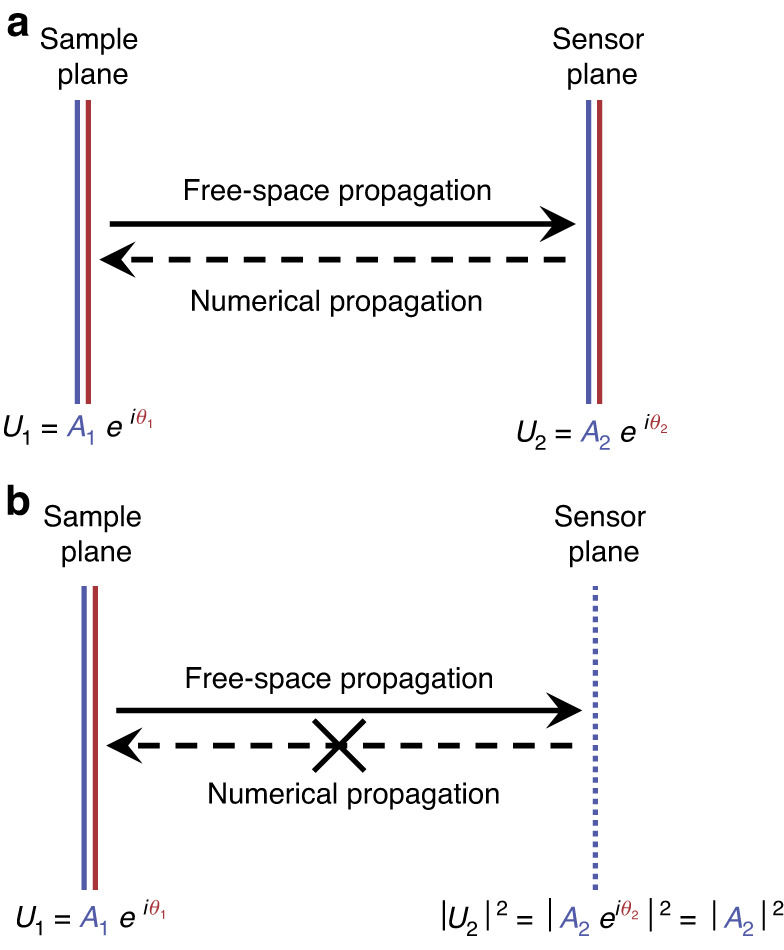
Calculating complex field at the sample plane from the complex field or the intensity at the sensor plane. **a** The complex field at the sample plane can be directly calculated from the complex field at the sensor plane. **b** The complex field at the sample plane cannot be directly calculated from the intensity at the sensor plane alone. *U*: complex field. *A*: amplitude. *θ*: phase

As is common in inverse problems, calculating the phase directly from an intensity measurement after propagation is usually ill-posed^7^. Suppose the complex field at the sensor plane is known. We can directly calculate the complex field at the sample plane using numerical propagation^8^ (Fig. 2a). However, in reality, the sensor only records the intensity but loses the phase, and, moreover, it is necessarily sampled by pixels of finite area size. Because of these complications, the complex field distribution at the sample plane generally cannot be calculated in a straightforward manner (Fig. 2b).

We can transform phase recovery into a well-posed/deterministic problem by introducing extra information, such as holography or interferometry at the expense of having to introduce a reference wave^8,9^, Shack-Hartmann wavefront sensing which introduces a microlens array at the conjugate plane^10,11^, and transport of intensity equation requiring multiple through-focus amplitudes^12,13^. Alternatively, we can solve this ill-posed phase recovery problem in an iterative manner by optimization, i.e., the so-called phase retrieval such as Gerchberg-Saxton-Fienup algorithm^14–16^, multi-height algorithm^17–19^, real-space ptychography^20–22^, and Fourier ptychography^23,24^. Next, we introduce these classical phase recovery methods in more detail.

### Holography/interferometry

By interfering the unknown wavefront with a known reference wave, the phase difference between the object wave and the reference wave is converted into the intensity of the resulting hologram/interferogram due to alternating constructive and destructive interference of the two waves across their fronts. This enables direct calculation of the phase from the hologram^8^.

**Fig. 3:**
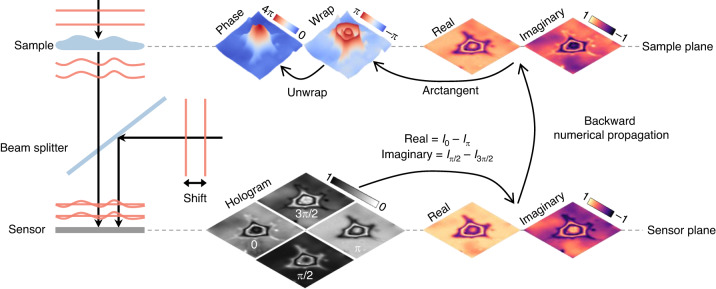
Description of in-line holography based on the four-step phase-shifting algorithm. *I*_0_: hologram with 0 phase delay. *I*_π/2_: hologram with π/2 phase delay. *I*_π_: hologram with π phase delay. *I*_3__π/__*2*_: hologram with 3π/2 phase delay

In in-line holography, where the object beam and the reference beam are along the same optical axis, four-step phase-shifting algorithm is commonly used for phase recovery (Fig. 3)^25^. At first, the complex field of the object wave at the sensor plane is calculated from the four phase-shifting holograms. Next, the complex field at the sample plane is obtained through numerical propagation. Then, by applying the arctangent function over the final complex field, a phase map in the range of (−π, π] is obtained, i.e., the so-called wrapped phase. The final sample phase is obtained after phase unwrapping. Other multiple-step phase-shifting algorithms are also possible for phase recovery^26^. Spatial light interference microscopy (SLIM), as a well-known QPI method, combines the phase-shifting algorithm with a phase contrast microscopy for phase recovery over transparent samples^27^.

**Fig. 4:**
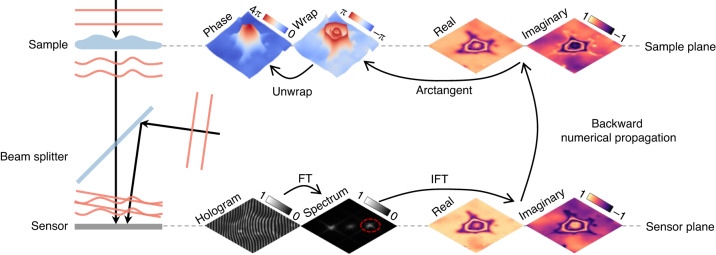
Description of off-axis holography based on spatial spectral filtering. FT Fourier transform, IFT inverse Fourier transform

In off-axis holography, where the reference beam is slightly tilted from the optical axis, the phase is modulated into a carrier frequency that can be recovered through spatial spectral filtering with only one holographic measurement (Fig. 4)^28^. By appropriately designing the carrier frequency, the baseband that contains the reference beam can be well separated from the object beam. After transforming the measured hologram into the spatial frequency domain through a Fourier transform (FT), one can select the +1st or −1st order beam and move it to the baseband. By applying an inverse FT, the object beam can be recovered. One has to be careful, however, not to exceed the Nyquist limit on the camera as the angle between reference and object increases. Moreover, as only a small part of the spatial spectrum is taken for phase recovery, off-axis holography typically wastes a lot of spatial bandwidth product of the system. To enhance the utilization of the spatial bandwidth product, the Kramers-Kronig relationship and other iterative algorithms have been recently applied in off-axis holography^29–31^.

Both the in-line and off-axis holography discussed above are lensless, where the sensor and sample planes are not mutually conjugated. Therefore, a backward numerical propagation from the former to the latter is necessary. The process of numerical propagation can be omitted if additional imaging components are added to conjugate the sensor and sample planes, such as digital holographic microscopy^32^.

### Shack-Hartmann wavefront sensing

**Fig. 5:**
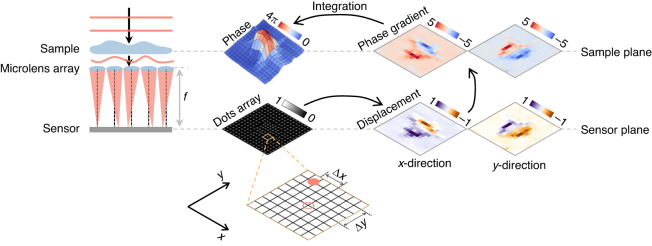
Description of the Shack-Hartmann wavefront sensor. *f*: focal length of microlens array

If we can obtain the horizontal and vertical phase gradients of a wavefront in some ways, then the phase can be recovered by integrating the phase gradients in these orthogonal directions. Shack-Hartmann wavefront sensor^10,11^ is a classic way to do so from the perspective of geometric optics. It usually consists of a microlens array and an image sensor located at its focal plane (Fig. 5). The phase gradient of the wavefront at the surface of each microlens is calculated linearly from the displacement of the focal point on the focal plane, in both horizontal and vertical (*x*-axis and *y*-axis) directions. The phase can then be computed by integrating the gradient at each point, whose resolution depends on the density of the microlens array. In addition, quantitative differential interference contrast microscopy^33^, quantitative differential phase contrast microscopy^34^, and quadriwave lateral shearing interferometry^35^ also recover the phase from its gradients. They may achieve higher resolution than the Shack-Hartmann wavefront sensor.

### Transport of intensity equation

**Fig. 6:**
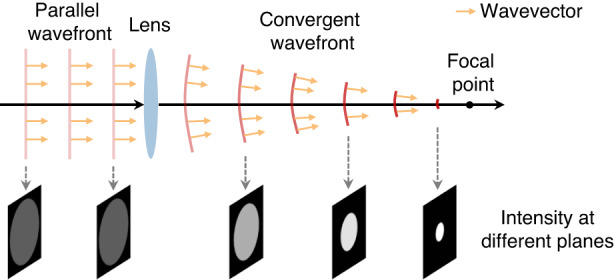
A convex lens converges light to a focal point

For a light field, the wavefront determines the axial variation of the intensity in the direction of propagation. Specifically, there is a quantitative relationship between the gradient and curvature of the phase and the axial differentiation of intensity, the so-called transport of intensity equation (TIE)^12^. This relationship has an elegant analogy to fluid mechanics, approximating the light intensity as the density of a compressible fluid and the phase gradient as the lateral pressure field^36^. TIE can be derived from three different perspectives: the Helmholtz equations in the paraxial approximation, and the Fresnel diffraction and Poynting theorem in the paraxial and weak-defocusing approximation^13^. The gradient and curvature of the phase together determine the wavefront shape, whose normal vector is then parallel to the wavevector at each point of the wavefront, and consequently to the direction of energy propagation. In turn, variations in the lateral energy flux also result in axial variations of the intensity. Convergence of light by a convex lens is an intuitive example (Fig. 6): the wavefront in front of the convex lens is a plane, whose wavevector is parallel to the direction of propagation. As such, the intensity distribution on different planes is constant; that is, the axial variation of the intensity is equal to zero. Then, the convex lens changes the wavefront so that all wavevectors are directed to the focal point, and therefore, as the light propagates, the intensity distribution becomes denser and denser, meaning that the intensity varies in the axial direction (equivalent, its axial derivative is not zero).

**Fig. 7:**
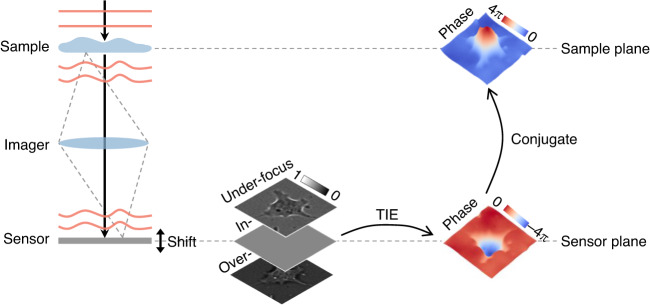
Description of phase recovery by transport of intensity equation (TIE)

As there is a quantitative relationship between the gradient and curvature of the phase and the axial differentiation of intensity, we can exploit it for phase recovery (Fig. 7). By shifting the sensor axially, intensity maps at different defocus distances are recorded, which can be used to approximate the axial differential by numerical difference, and thus calculate the phase through TIE. Due to the addition of the imager, the sensor and sample planes are conjugated. Besides, TIE can also be used in lensless systems to recover the phase at the defocus plane, which thus requires an additional numerical propagation^13^.

It is worth noting that TIE is suitable for a complete and partially coherent light source, and the resulting phase is continuous and does not require phase unwrapping, while it is only effective in the case of paraxial and weak-defocusing approximation^13^.

### Phase retrieval

**Fig. 8:**
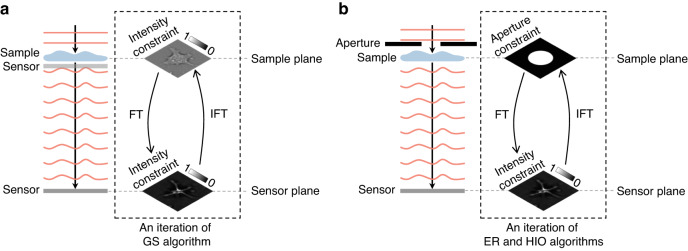
Description of alternating-projection algorithms. **a** Gerchberg-Saxton algorithm. **b** Error reduction and hybrid input-output algorithms

If extra information is not desired to be introduced, then calculating the phase directly from a propagated intensity measurement is an ill-posed problem. We can overcome such difficulty through incorporating prior knowledge. This is also known as regularization. In the Gerchberg-Saxton (GS) algorithm^14^, the intensity at the sample plane and the far-field sensor plane recorded by the sensor are used as constraints. A complex field is projected forward and backward between these two planes using the Fourier transform and constrained by the intensity iteratively; the resulting complex field will gradually approach a solution (Fig. 8a). Fienup changed the intensity constraint at the sample plane to the aperture (support region) constraint, so that the sensor only needs to record one intensity map, resulting in the error reduction (ER) algorithm and the hybrid input-output (HIO) algorithm (Fig. 8b)^15,16^. In addition to the aperture constraint, one can introduce other physical constraints such as histogram^37^, atomicity^38^, and absorption^39^ to reduce the ill-posedness of phase retrieval. Furthermore, many types of sparsity priors such as spatial domain^40^, gradient domain^41,42^, and wavelet domain^43^ are effective regularizers for phase retrieval.

**Fig. 9:**
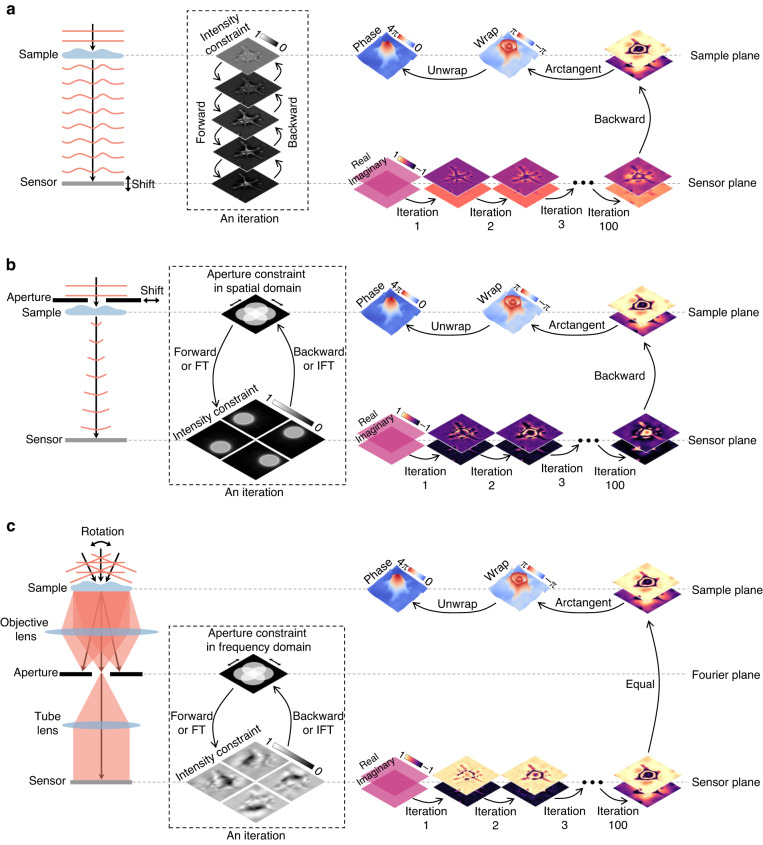
Description of multi-intensity alternating projection. **a** Axial multi-intensity alternating projection. **b** Radial multi-intensity alternating projection. **c** Angular multi-intensity alternating projection. Forward: forward numerical propagation. Backward: backward numerical propagation

Naturally, if more intensity maps are recorded by the sensor, there will be more prior knowledge for regularization, further reducing the ill-posedness of the problem. By moving the sensor axially, the intensity maps of different defocus distances are recorded as an intensity constraint, and then the complex field is computed iteratively like the GS algorithm (Fig. 9a), the so-called multi-height phase retrieval^17–19^. In this axial multi-intensity alternating projection method, the distance between the sample plane and the sensor plane is usually kept as close as possible, so that numerical propagation is used for projection instead of Fourier transform. Meanwhile, with a fixed position of the sensor, multiple intensity maps can also be recorded by radially moving the aperture near the sample, and then the complex field is recovered iteratively like the ER and HIO algorithms (Fig. 9b), the so-called real-space ptychography^20–22^. In this radial multi-intensity alternating projection method, each adjoining aperture constraint overlaps one another and expands the field of view in real space. Furthermore, angular multi-intensity alternating projection is also possible. By switching the aperture constraint from the spatial domain to the frequency domain with a lens system, multiple intensity maps with different frequency information are recorded by changing the angle of the incident light (Fig. 9c), the so-called Fourier ptychography^23,24^. Due to the change of illumination angle, high-frequency information that originally exceeds the numerical aperture is recorded, expanding the Fourier bandwidth in reciprocal space. Recently, synthetic aperture ptychography^44^ was proposed to simultaneously expand the bandwidth in real space and reciprocal space, in which an extended plane wave is used to illuminate a stationary object and subsequently a coded image sensor is translated within the far field to record data.

In addition to alternating projections, there are two most representative non-convex optimization methods, namely the Wirtinger flow^45^ and truncated amplitude flow algorithms^46^. They can be transformed into convex optimization problems through semidefinite programming, such as the PhaseLift algorithm^47^.

### Recovery of low-frequency phase component

As mentioned at the beginning, because the phase information of the light field is converted into amplitude variations during propagation, one can recover the phase from the recorded amplitude distribution. However, low-frequency phase component causes less amplitude variations, which is difficult for detection. A more quantitative analysis can be performed through the phase transfer function^13^, which characterizes the transfer response of phase content at different spatial frequencies for an imaging system. For holography and Shack-Hartmann wavefront sensing, due to the interference phenomenon or the microlens array, the low-resolution phase component is converted into a fringe pattern or focus translation, which can be easily detected. For other lensless methods of recovering phase from propagation intensity maps, such as lensless TIE, Gerchberg-Saxton-Fienup algorithm, multi-height algorithm, and real-space ptychography with an unknown probe beam, their phase transfer function of the low-frequency component is close to zero. That is to say, the slow-varying phase gradient cannot induce sufficient intensity contrast to be detected and thus cannot be recovered through subsequent algorithms. Coded ptychography^48^ is an effective solution, in which the coded layer (such as disorder-engineered surface^49^ or fixed blood-cell layer^50,51^) effectively converts the phase information of different spatial frequencies into detectable distortions in the diffraction patterns. Similarly, the coded layer can also be used in the multi-height algorithm to recover the slow-varying phase profiles^52^. As for the lens-based case, such as lens-based TIE^53,54^, Fourier ptychography^55^, and quantitative differential phase contrast microscopy^56^, the phase transfer function of the imaging system can be modulated by changing the illumination angle, thereby collecting more low-frequency phase information.

### Deep learning (DL) for phase recovery

**Fig. 10:**
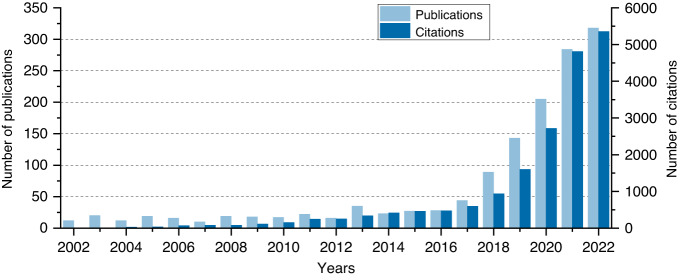
Growth in interest in using “deep learning for phase recovery” overtime is depicted by the number of publications and citations on Web of Science. The used search code is “*TS* = *((“phase recovery” OR “phase retrieval” OR “phase imaging” OR “holography” OR “phase unwrapping” OR “holographic reconstruction” OR “hologram” OR “fringe pattern”) AND (“deep learning” OR “network” OR “deep-learning”))*”

In recent years, as an important step towards true artificial intelligence (AI), deep learning^57^ has achieved unprecedented performance in many tasks of computer vision with the support of graphics processing units (GPUs) and large datasets. Similarly, since it was first used to solve the inverse problem in imaging in 2016^58^, deep learning has demonstrated promising potential in the field of computational imaging^59^. In the meantime, there is a rapidly growing interest in using deep learning for phase recovery (Fig. 10).

**Fig. 11:**
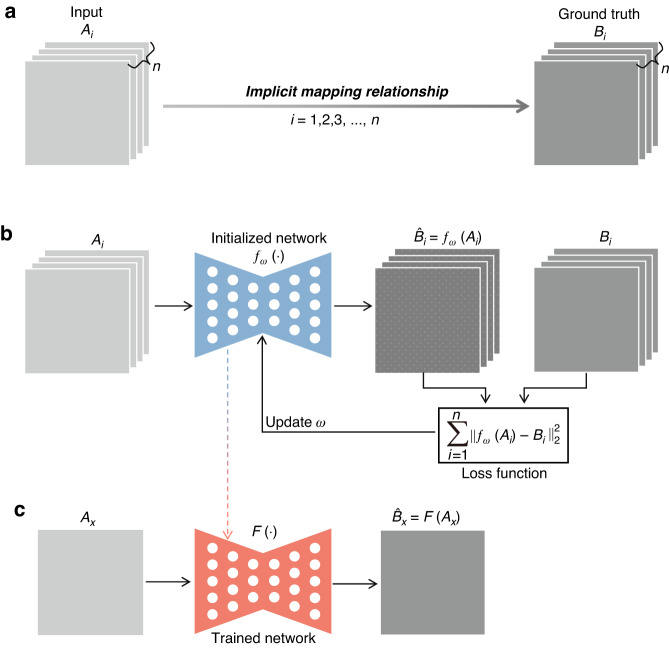
Implementation of deep learning with paired dataset and supervised learning. **a** Datasets collection. **b** Network training. **c** Inference via a trained network. *ω* the parameters of the neural network, *n* the sample number of the training dataset

For the vast majority of “DL for PR”, the implementation of deep learning is based on the training and inference of artificial neural networks (ANNs)^60^ through input-label paired dataset, known as supervised learning (Fig. 11). In view of its natural advantages in image processing, the convolutional neural network (CNN)^61^ is the most widely used ANN for phase recovery. Specifically, in order for the neural network to learn the mapping from physical quantity *A* to *B*, a large number of paired examples need to be collected to form a training dataset that implicitly contains this mapping relationship (Fig. 11a). Then, the gradient of the loss function is propagated backward through the neural network, and the network parameters are updated iteratively, thus internalizing this mapping relationship (Fig. 11b). After training, the neural network is used to infer *B*_*x*_ from an unseen *A*_*x*_ (Fig. 11c). In this way, deep learning has been used in all stages of phase recovery and phase processing.

**Fig. 12:**
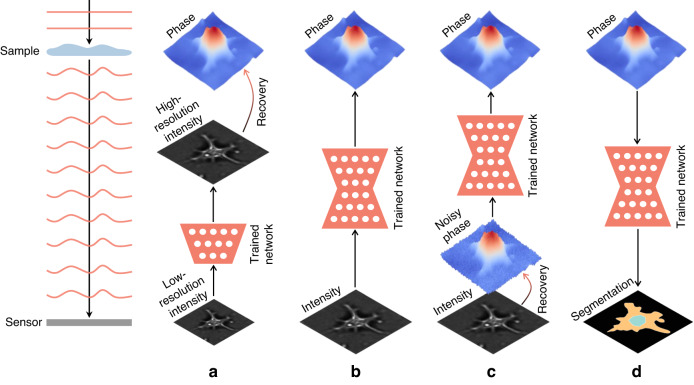
Overview example of “deep learning (DL) for phase recovery (PR) and phase processing”. **a** DL-pre-processing for PR. **b** DL-in-processing for PR. **c** DL-post-processing for PR. **d** DL for phase processing
- In the section “DL-in-processing for phase recovery”, the neural network directly performs phase recovery (Fig. 12b) or participates in the process of phase recovery together with the physical model or physics-based algorithm by supervised or unsupervised learning modes.
- In the section “DL-post-processing for phase recovery”, the neural network performs post-processing after phase recovery, such as noise reduction (Fig. 12c), resolution enhancement, aberration correction, and phase unwrapping.
- In the section “Deep learning for phase processing”, the neural network uses the recovered phase for specific applications, such as segmentation (Fig. 12d), classification, and imaging modal transformation.

In fact, the rapid pace of deep-learning-based phase recovery has been documented in several excellent review papers. For example, Barbastathis et al.^59^ and Rivenson et al.^62^ reviewed how supervised deep learning powers the process of phase retrieval and holographic reconstruction. Zeng et al.^63^ and Situ et al.^64^ mainly focused on the use of deep learning in digital holography and its applications. Zhou et al.^65^ and Wang et al.^66^ reviewed and compared different usage strategies of AI in phase unwrapping. Dong et al.^67^ introduced a unifying framework for various algorithms and applications from the perspective of phase retrieval and presented its advances in machine learning. Park et al.^68^ discussed AI-QPI-based analysis methodologies in the context of life sciences. Differently, depending on where the neural network is used, we review various methods from the following four perspectives:

- In the section “DL-pre-processing for phase recovery”, the neural network performs some pre-processing on the intensity measurement before phase recovery, such as pixel super-resolution (Fig. 12a), noise reduction, hologram generation, and autofocusing.

Finally, we summarize how to effectively use deep learning in phase recovery and look forward to potential development directions (see the section “Conclusion and outlook”). To let readers learn more about phase recovery, we present a live-updating resource (https://github.com/kqwang/phase-recovery).

## DL-pre-processing for phase recovery

A summary of “DL-pre-processing for phase recovery” is presented in Table 1 and is described below, including the “Pixel super-resolution”, “Noise reduction”, “Hologram generation”, and “Autofocusing” sections.

**Table 1:** Summary of “DL-pre-processing for phase recovery”

Task	Reference	Input	Output	Network	Training dataset	Loss function
Pixel Super-resolution	Luo et al.^70^	Sub-pixel LR holograms	HR hologram	U-Net	Expt. and Sim.: 1600 pairs	SSIM
	Byeon et al.^74^	LR hologram	HR hologram	SRCNN	Sim.: 192 pairs	*l*_2_-norm
	Xin et al.^75^	LR hologram	HR hologram	Fast SRCNN	Sim.: 5000 pairs	*l*_2_-norm
	Ren et al.^76^	LR hologram	HR hologram	ResNet and SubPixelNet	Expt.: 800 pairs	*l*_2_-norm
Noise reduction	Yan et al.^80^	Noisy fringe pattern	Noise-free fringe pattern	DnCNN	Sim.: 80,000 pairs	*l*_1_-norm
	Lin et al.^81^	Noisy fringe pattern	Noise-free fringe pattern	CNN	Sim.: 230,400 pairs	*l*_2_-norm
	Hao et al.^83^	Noisy fringe sub-pattern	Noise-free fringe sub-pattern	FFDNet	Sim.: 1200 pairs	*l*_2_-norm
	Zhou et al.^84,85^	Noisy fringe pattern	Noise-free fringe pattern	Spectral CNN	Sim.: 1200 pairs	---
	Reyes-Figueroa et al.^86^	Noisy fringe pattern	Noise-free fringe pattern	U-Net and ResNet	Sim.: 25,000 pairs	*l*_1_-norm
	Gurrola-Ramos et al.^87^	Noisy fringe pattern	Noise-free fringe pattern	U-Net and DenseNet	Sim.: 1500 pairs	*l*_1_-norm
Hologram generation	Zhang et al.^88,89^	Hologram	Phase-shifting holograms	Y-Net	Sim.: ---	*l*_2_-norm
	Yan et al.^91^	Hologram	Single phase-shifting hologram	ResNet	Sim: 12,000 pairs	GAN loss
	Zhao et al.^92^	Hologram	Phase-shifting holograms	MPRNet	Sim.: ---	Charbonnier and Edge
	Huang et al.^94^	Hologram	Phase-shifting holograms	Y-Net	Expt.: 4000 pairs	*l*_2_-norm
	Wu et al.^95^	Hologram	Single phase-shifting hologram	U-Net	Sim.: 6400 pairs	GAN loss
	Luo et al.^96^	Hologram	Multi-distance holograms	U-Net	Sim.: 440 pairs	GAN loss
	Li et al.^97^	Hologram	Hologram with another wavelength	U-Net	Sim.: 20,000 pairs	GAN loss
	Li et al.^98^	Two holograms	Hologram with another wavelength	Y-Net	Sim.: 8000 pairs	*l*_1_-norm
	Xu et al.^99^	dual-wavelength hologram	Two single-wavelength holograms	U-Net	Sim.: 1800 pairs	*l*_2_-norm
Autofocusing	Pitkäaho et al.^100^	Hologram	Defocus distance (21 types)	AlexNet	Expt. and sim.: 485,856 pairs	Cross entropy
	Ren et al.^101^	Hologram	Defocus distance (5 types)	CNN	Expt.: >5000 pairs	Cross entropy
	Son et al.^102^	Hologram	Defocus distance (10 types)	CNN	Sim.: 40,180 pairs	Cross entropy
	Couturier et al.^103^	Hologram	Defocus distance (101 types)	DenseNet	Expt.: 7000 pairs	Cross entropy
	Ren et al.^104^	Hologram (amplitude or phase object)	Defocus distance	CNN	Expt.: 5000 and 2000 pairs	*l*_1_-norm
	Pitkäaho et al.^105^	Hologram (cells)	Defocus distance	AlexNet and VGG	Expt.: 437,271 pairs	*l*_2_-norm
	Jaferzadeh et al.^106^ and Moon et al.^107^	Hologram (single cell)	Defocus distance	CNN	Expt.: 3000 and 2400 pairs	*l*_2_-norm
	Tang et al.^111^	Fixed vector	Defocus distance	MLP (untrained)	Expt.: 1	*l*_2_-norm
	Cuenat et al.^108^	Hologram (USAF 1951)	Defocus distance	Vision Transformer	Expt.: 104,400 pairs Sim.: 40,000 pairs	log cosh
	Lee et al.^109^	Spatial spectrum	Defocus distance	CNN	Sim.: ---	*l*_2_-norm
	Shimobaba et al.^110^	1/4 power spectrum	Defocus distance	CNN	Sim.: ---	*l*_2_-norm

“---” indicates not available. “GAN loss” means training the network in an adversarial generative way

*LR* low-resolution, *HR* high-resolution, *Expt.* experiment, *Sim.* simulation, *MLP* multi-layer perceptron

### Pixel super-resolution

A high-resolution image generally reveals more detailed information about the object of interest. Therefore, it is desirable to recover a high-resolution image from one or multiple low-resolution measurements of the same field of view, a process known as pixel super-resolution. Similarly, from multiple sub-pixel-shifted low-resolution holograms, a high-resolution hologram can be recovered by pixel super-resolution algorithms^69^. Luo et al.^70^ proposed to use the U-Net for this purpose. Compared with iterative pixel super-resolution algorithms, this deep learning method has an advantage in inference time while ensuring the same level of resolution improvement. It maintains high performance even with a reduced number of input low-resolution holograms.

**Fig. 13:**
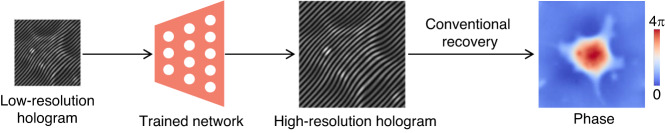
Description of deep-learning-based hologram super-resolution

After the pixel super-resolution CNN (SRCNN) was proposed for single-image super-resolution in the field of image processing^71^, this type of deep learning method was also used in other optical super-resolution problems, such as bright-field microscopy^72^ and fluorescence microscopy^73^. Similarly, this method of inferring corresponding high-resolution images from low-resolution versions via deep neural networks can also be used for holograms pixel super-resolution before doing phase recovery by conventional recovery methods (Fig. 13).

Byeon et al.^74^ first applied the SRCNN to hologram pixel super-resolution, and named it HG-SRCNN. Compared with conventional focused-image-trained SRCNN and bicubic interpolation, this method, trained with defocus in-line holograms, can infer higher-quality high-resolution holograms. Xin et al.^75^ used an improved fast SRCNN (FSRCNN) to do pixel super-resolution for white-light holograms, significantly improving the identification and accuracy of three-dimensional (3D) measurement results. Under the premise of improved accuracy, the inference speed of FSRCNN is nearly ten times faster than that of SRCNN.

Ren et al.^76^ proposed to use a CNN, incorporating the residual network (ResNet) and sub-pixel network (SubPixelNet), for pixel super-resolution of a single off-axis hologram. They found that compared to *l*_1_-norm and structural similarity index (SSIM)^77^, the neural network trained using *l*_2_-norm as the loss function performed best. Moreover, this deep learning method reconstructs high-resolution off-axis holograms with better quality than conventional image super-resolution methods, such as bicubic, bilinear, and nearest-neighbor interpolations.

### Noise reduction

Most phase recovery methods, especially holography, are performed with a coherent light source; therefore, coherent noise is unavoidable. In addition, noise can be caused by environmental disturbances and the recording process of the image sensor. Therefore, reducing the noise from the hologram before phase recovery is essential. Filter-based methods, such as windowed Fourier transform (WFT)^78^, have been widely used in hologram noise reduction, but most of these methods face a trade-off between good filtering performance and time cost.

**Fig. 14:**
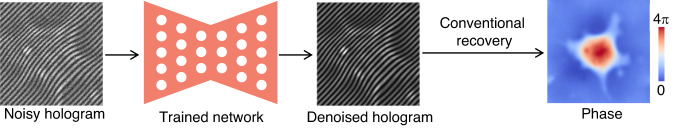
Description of deep-learning-based hologram noise reduction

In 2017, Zhang et al.^79^ opened the door to image denoising using the deep CNN, called DnCNN. Subsequently, the DCNN was introduced to the field of fringe analysis for fringe pattern denoising (Fig. 14).

Yan et al.^80^ first applied the DnCNN to fringe pattern denoising, which has higher precision around image boundaries and needs less inference time than WFT. Similar conclusions can also be seen in the work of Lin et al.^81^. Then, inspired by the FFDNet^82^, Hao et al.^83^ downsampled the input fringe pattern into four sub-images before using the DnCNN for denoising, leading to a faster inference speed. Furthermore, Zhou et al.^84,85^ converted this batch-denoising DnCNN into the frequency domain. Specifically, they first computed the Fourier transform of the downsampled sub-images, then used the DnCNN to achieve noise reduction in the frequency domain, and finally applied upsampling and inverse Fourier transform to obtain the denoised fringe pattern. From the comparison results, their method outperforms that of Yan et al. and Hao et al. at different noise levels. Reyes-Figueroa et al.^86^ further showed that the U-Net and its improved version (V-Net) are better than DnCNN for fringe pattern denoising, because their proposed V-Net has more channels on the outer side than on the inner side, retaining more details. Given the U-Net’s outstanding mapping capabilities, Gurrola-Ramos et al.^87^ also improved it for fringe pattern denoising, where dense blocks are leveraged for reusing feature layers, local residual learning is used to address the vanishing gradient problem, and global residual learning is used to estimate the noise of the image instead of the denoised image directly. Compared with other neural networks mentioned above, it has a minor model complexity while maintaining the highest accuracy.

### Hologram generation

**Fig. 15:**
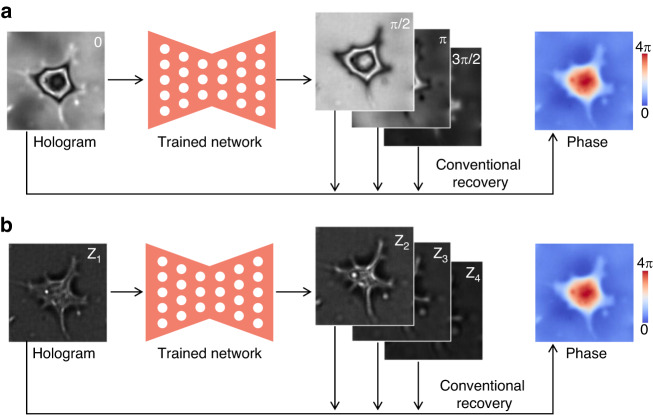
Description of deep-learning-based hologram generation. **a** Phase-shifting method. **b** Axial multi-intensity alternating projection method

As mentioned in the Introduction, in order to recover the phase, multiple intensity maps are needed in many cases, such as phase-shifting holography and axial multi-intensity alternating projection. Given its excellent mapping capability, the neural network can be used to generate other relevant holograms from known ones, thus enabling phase recovery that requires multiple holograms (Fig. 15). In this approach, the input and output usually belong to the same imaging modality with high feature similarity, so it is easier for the neural network to learn. Moreover, the dataset is collected only by experimental record or simulation generation, without the need for phase recovery as ground truth in advance by conventional methods.

Zhang et al.^88,89^ first proposed the idea of generating holograms with holograms before phase recovery with the conventional method (Fig. 15a). From a single hologram, the other three holograms with π/2, π, and 3π/2 phase shifts were simultaneously generated by the Y-Net^90^, and then phase recovery was implemented by the four-step phase-shifting method. The motivation to infer holograms instead of phase via a network is that for different types of samples, the spatial differences between their holograms were significantly lower than that of their phase. Accordingly, this phase recovery based on the hologram generation has better generalization ability than recovering phase from holograms directly with the neural network, especially when the spatial characteristics differences of the phase between the training and testing datasets are relatively large^89^. Since the phase-shift between the generated holograms is equal, Yan et al.^91^ proposed to generate noise-free phase-shifting holograms using a simple end-to-end generative adversarial network (GAN) in a manner of sequential concatenation. Subsequently, for better performance in balancing spatial details and high-level semantic information, Zhao et al.^92^ applied the multi-stage progressive image restoration network (MPRNet)^93^ for phase-shifting hologram generation. Huang et al.^94^ and Wu et al.^95^ then expanded this approach from four-step to three-step and two-step phase-shifting methods, respectively.

Luo et al.^96^ proposed to generate holograms with different defocus distances from one hologram via a neural network, and then achieve phase recovery with alternating projection (Fig. 15b). Similar to the work of Zhang et al.^89^, they proved that the use of neural networks with less difference between the source domain and the target domain could enhance the generalization ability. As for multi-wavelength holography, Li et al.^97,98^ harnessed a neural network to generate a hologram of another wavelength from one or two holograms of known wavelength, thereby realizing two-wavelength and three-wavelength holography. At the same time, Xu et al.^99^ realized a one-shot two-wavelength and three-wavelength holography by generating the corresponding single-wavelength holograms from a two-wavelength or three-wavelength hologram with information crosstalk.

### Autofocusing

In lensless holography, the phase of the sample plane can only be recovered if the distance between the sensor plane and the sample plane is known. Defocus distance estimation thus becomes a fundamental problem in holography, which is also known as autofocusing.

**Fig. 16:**
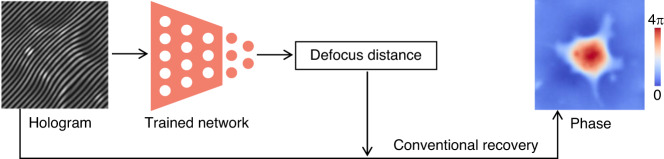
Description of deep-learning-based hologram numerical refocusing

Deep learning methods for autofocus essentially use the neural network to estimate the defocus distance from the hologram (Fig. 16), which can be regarded as either a classification problem^100–103^ or a regression problem^104–110^.

From the perspective of classification, Pitkäaho et al.^100^ first proposed to estimate the defocus distance from the hologram by a CNN. In their scheme, the zero-order and twin-image terms need to be removed before the trained neural network classifies the holograms into different discrete defocus distances. Meanwhile, Ren et al.^101^ advocate directly using raw holograms collected at different defocus distances as the input of the neural networks. Furthermore, they revealed the advantages of neural networks over other machine learning algorithms in the task of autofocusing. Immediately afterward, Son et al.^102^ also verified the feasibility of autofocus by classification through numerical simulations. Subsequently, Couturier et al.^103^ improved the accuracy of defocus distance estimation by using a deeper CNN for categorizing defocus distance into a greater number of classes.

Nevertheless, no matter how many classes there are, the defocus distance estimated by these classification-based methods is also discrete, which is still not precise enough in practice. Thus, Ren et al.^104^ further developed an approach to treat the defocus distance estimation as a regression problem, where the output of the neural network is continuous. They verified the superiority of this deep-learning-based regression method with amplitude samples and phase samples, respectively, and tested the adaptability under different exposure times and incident angles. Later, Pitkäaho et al.^105^ also extended their previous classification-based work^100^ to this regression-based approach. While these methods estimate the defocus distance of the entire hologram, Jaferzadeh et al.^106^ and Moon et al.^107^ proposed to take out the region of interest from the whole hologram as the input to estimate the defocus distance. In order to get rid of the constraint of known defocus distance as the label of the training dataset, Tang et al.^111^ proposed to iteratively infer the defocus distance by an untrained network with a defocus hologram and its in-focus phase. Later on, Cuenat et al.^108^ demonstrated the superiority of the Vision Transformer^112^ over typical CNNs in defocus distance estimation. Because the spatial spectrum information is also helpful for the defocus distance estimation^113^, Lee et al.^109^ and Shimobaba et al.^110^ proposed to use the spatial spectrum or power spectrum of holograms as the network input to estimate the defocus distance.

## DL-in-processing for phase recovery

In “DL-in-processing for phase recovery”, the neural network directly performs the inference process from the measured intensity image to the phase (see the “Network-only strategy” section), or together with the physical model or physics-based algorithm to achieve the inference (see the “Network-with-physics strategy” section).

### Network-only strategy

The network-only strategy uses a neural network to perform phase recovery, where the network input is the measured intensity image and the output is the phase. A summary of various methods is presented in Table 2 and described below, where we classify them into dataset-driven (DD) and physics-driven (PD) approaches.

**Table 2:** Summary of network-only strategy

Task	Reference	Input	Output	Network	Training dataset	Loss function
Dataset-driven (DD) approach	Sinha et al.^114^	Diffraction image	Phase	U-Net and ResNet	Expt.: 10,000 pairs	*l*_1_-norm
	Li et al.^115^	Diffraction image	Phase	U-Net and ResNet	Expt.: 10,000 pairs	NPCC
	Deng et al.^117^	Diffraction image	Phase	U-Net and ResNet	Expt.: 10,000 pairs	NPCC
	Goy et al.^118^	Weak-light diffraction	Phase	U-Net and ResNet	Expt.: 9500 pairs	NPCC
	Wang et al.^119^	In-line hologram	Phase	U-Net and ResNet	Expt.: 9000 and 11,623 pairs	*l*_2_-norm
	Nguyen et al.^120^	Multiple LR intensity images (Fourier ptychography)	HR phase	U-Net and DenseNet	Expt.: ---	GAN loss and *l*_1_-norm
	Cheng et al.^121^	LR intensity image (Fourier ptychography)	HR phase and amplitude	CNN and ResNet	Expt.: 20 fields-of-view	*l*_2_-norm
	Cherukara et al.^122^	Far-field diffraction	Phase or amplitude	SegNet (two)	Sim.: 180,000 pairs	Cross-entropy
	Ren et al.^123^	Off-axis hologram	Phase or amplitude	ResNet and SubPixelNet	Expt.: >10,000 pairs	*l*_2_-norm
	Yin et al.^124^	Hologram	Phase	U-Net	Expt.: 2400 and 200–2000 (unpaired)	Cycle-GAN loss
	Lee et al.^125^	Hologram	Phase and amplitude	U-Net and CNN	Expt.: 600–9060 (unpaired)	Cycle-GAN loss and SSIM
	Hu et al.^126^	Spots’ intensity image	Phase	U-Net and ResNet	Sim.: 46,080 pairs	*l*_2_-norm
	Wang et al.^127^	Defocus intensity image	Phase	U-Net and ResNet	Expt.: 20,037 pairs	*l*_2_-norm
	Zhou et al.^128^	LR defocus intensity image	HR phase	U-Net	Expt.: 1300 pairs	*l*_2_-norm
	Pirone et al.^129^	Hologram in different angles	Phase	CAN	Expt.: 4000 pairs	*l*_1_-norm
	Chang et al.^130^	Diffraction image (Electron)	Phase	U-Net and ResNet	Sim.: 250,000 pairs	*l*_1_-norm
	Xue et al.^132^	Bright- and dark-field images	Phase	U-Net and BNN	Expt.: 185 groups	*l*_1_-norm and uncertainty term
	Li et al.^133^	Two images of symmetric illumination	Phase	U-Net	Sim.: 1301 groups	GAN loss
	Wang et al.^90,134^	Hologram	Phase and amplitude	Y-Net	Expt.: 1331 pairs	*l*_2_-norm
	Zeng et al.^135^	Hologram	Phase or amplitude	CapsNet	Expt.: ---	*l*_2_-norm
	Wu et al.^136^	Far-field diffraction	Phase and amplitude	Y-Net	Sim.: 142,500 groups	Loss in real and reciprocal space
	Huang et al.^137^	Two or 3 holograms	Complex field	U-Net and Recurrent CNN	Expt.: 208 groups	GAN loss and *l*_1_-norm and SSIM
	Uelwer et al.^138^	Far-field diffraction	Phase	Cascaded neural network	Sim.: ---	*l*_2_-norm or *l*_1_-norm
	Castaneda et al.^139^	Off-axis hologram	Wrapped phase	U-Net	Expt.: 1512 pairs	GAN loss and TSM and STD
	Jaferzadeh et al.^140^	Off-axis hologram	Phase	U-Net	Expt.: 900 pairs	GAN loss
	Luo et al.^141^	Hologram	Phase	MCN	Expt.: 1 pair	Bucket error rate (BER) loss
	Ding et al.^142^	LR image	HR phase	U-Net and Swin Transformer	Expt.: 3500 and 3500 (unpaired)	Cycle-GAN loss
	Ye et al.^144^	Far-field diffraction	Complex field	MLP and CNN	Sim. and Expt.: ---	*l*_1_-norm
	Chen et al.^145,146^	Three or 4 holograms	Complex field	ResNet and Fourier module (FIN)	Expt.: 600 groups	*l*_1_-norm, complex domain and perceptual loss
	Shu et al.^147^	Hologram	Phase	Network based on NAS	Expt.: 276 pairs	MixGE and binary and sparsity loss
Physics-driven (PD) approach	Boominathan et al.^149^	LR intensity images (Fourier ptychography)	HR Phase and amplitude	U-Net	Sim.: 1 (input only)	*l*_2_-norm with physical model
	Wang et al.^150^	Diffraction image	Phase	U-Net	Sim. and Expt.: 1 (input only)	*l*_2_-norm with physical model
	Zhang et al.^151^	Diffraction image	Phase	U-Net	Sim. and Expt.: 1 (input only)	*l*_2_-norm with defocus distance and physical model
	Yang et al.^152,153^	Diffraction image	Phase and amplitude	U-Net	Sim. and Expt.: 1–180 (input only)	*l*_2_-norm with aperture constraint and physical model
	Bai et al.^154^	Hologram	dual-wavelength Phase	CDD	Expt.: 1 (input only)	*l*_2_-norm with physical model
	Galande et al.^155^	Hologram	Phase and amplitude	U-Net	Expt.: 1 (input only)	*l*_2_-norm with physical model and denoiser
	Yao et al.^159^	3D diffraction image	Phase and amplitude	3D Y-Net	Sim.: 52,000 (input only)	*l*_2_-norm with physical model
	Li et al.^160^	Two diffraction images	Phase	Two-to-one Y-Net	Sim.: 500 (input only)	*l*_2_-norm with physical model
	Bouchama et al.^161^	LR intensity images (Fourier ptychography)	HR Phase and amplitude	U-Net	Sim.: 10,000 (input only)	*l*_2_-norm with physical model
	Huang et al.^162^	Two holograms	Phase and amplitude	GedankenNet	Sim.: 100,000 (input only)	*l*_2_-norm and Fourier-domain *l*_1_-norm

“---” indicates not available

#### Dataset-driven approach

**Fig. 17:**
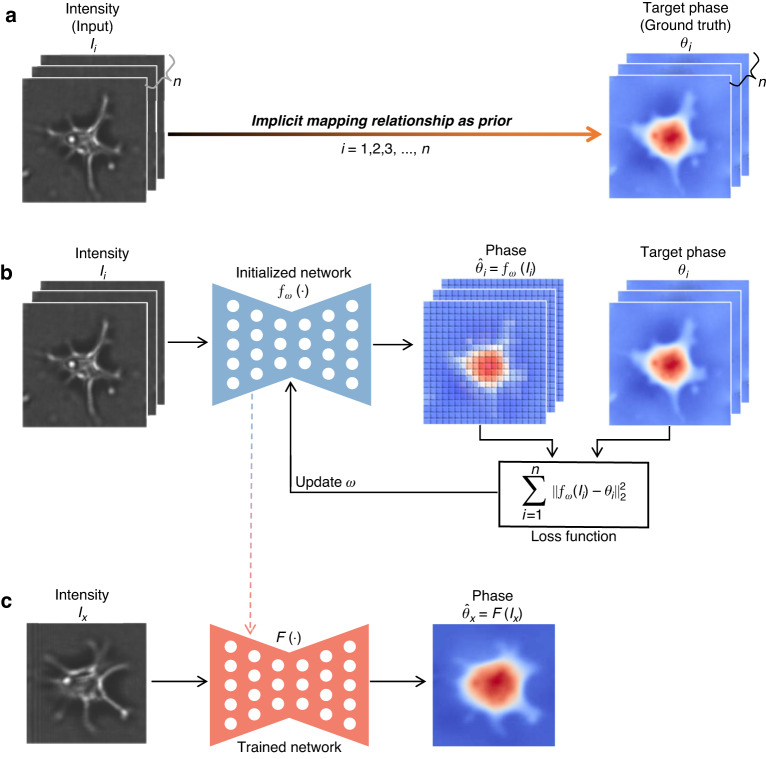
Description of dataset-driven network-only phase recovery. **a** Dataset collection. **b** Network training. **c** Inference via a trained network

As a supervised learning mode, data-driven deep learning phase recovery methods presuppose a large number of paired input-label datasets. Usually, it is necessary to experimentally collect a significant number of intensity images (such as diffraction images or holograms) as input, and use conventional methods to calculate the corresponding phase as ground truth (Fig. 17a). The key lies in that this paired dataset implicitly contains the mapping relationship from intensity to phase. Then, an untrained/initialized neural network is iteratively trained with the paired dataset as an *implicit prior*, where the gradient of the loss function propagates into the neural network to update the parameters (Fig. 17b). After training, the network is used as an end-to-end mapping to infer the phase from intensity (Fig. 17c). Therefore, the DD approach is to guide/drive the training of the neural network with this implicit mapping, which is internalized into the neural network as the parameters are iteratively updated.

Sinha et al.^114^ were among the first to demonstrate this end-to-end deep learning strategy for phase recovery, in which the phase of objects is inferred from corresponding diffraction images via a trained deep neural network. In dataset collection, they used a phase-only spatial light modulator (SLM) to load different public image datasets to generate the phase as ground truth, and after a certain distance, place the image sensor to record the diffraction image as input. The advantage is that both the diffraction image and the phase are known and easily collected in large quantities. Through comparative tests, they verified the adaptability of the deep neural network to unseen types of datasets and different defocus distances. Although this scheme cannot be used in practical application due to the use of the phase-type spatial light modulator, their pioneering work opens the door to deep-learning-inference phase recovery. For instance, Li et al.^115^ introduced the negative Pearson correlation coefficient (NPCC)^116^ as a loss function to train the neural network, and enhanced the spatial resolution by a factor of two by flattening the power spectral density of the training dataset. Deng et al.^117^ found that the higher the Shannon entropy of the training dataset, the stronger the generalization ability of the trained neural network. Goy et al.^118^ extended the work to phase recovery under weak-light illumination.

Meanwhile, Wang et al.^119^ extended the diffraction device of Sinha et al.^114^ to an in-line holographic device by adding a coaxial reference beam, and used the in-line hologram instead of the diffraction image as the input to a neural network for phase recovery. Nguyen et al.^120^ applied this end-to-end strategy for Fourier ptychography, inferring the high-resolution phase from a series of low-resolution intensity images via a U-Net, and Cheng et al.^121^ further used a single low-resolution intensity image under optimized illumination as the neural network input. Cherukara et al.^122^ extended this end-to-end deep learning strategy to CDI, in which they trained two neural networks with simulation datasets to infer the amplitude or phase of objects from far-field diffraction intensity maps, respectively. Ren et al.^123^ demonstrated the time and accuracy superiority of this end-to-end deep learning strategy over conventional numerical algorithms in the case of off-axis holography. Yin et al.^124^ introduced the cycle-GAN to extend this end-to-end deep learning strategy to the application scenario of unpaired datasets. Lee et al.^125^ replaced the forward generator of the cycle-GAN by numerical propagation, improving the phase recovery robustness of neural networks in highly perturbative configurations. Hu et al.^126^ applied this end-to-end deep learning strategy to the Shack-Hartmann wavefront sensor, inferring the phase directly from a spot intensity image after the microlens array. Wang et al.^127^ extended this end-to-end deep learning strategy to TIE, using a trained neural network to infer the phase of the cell object from a defocus intensity image illuminated by partially coherent light. Further, Zhou et al.^128^ used neural networks to infer high-resolution phase from a low-resolution defocus intensity image. Pirone et al.^129^ applied this hologram-to-phase deep learning strategy to improve the reconstruction speed of 3D optical diffraction tomography (ODT) from tens of minutes to a few seconds. Chang et al.^130^ expanded the illumination source from photons to electrons, recovering the phase images from electron diffraction patterns of twisted hexagonal boron nitride, monolayer graphene, and Au nanoparticles. Tayal et al.^131^ demonstrated the use of data augmentation and a symmetric invariant loss function to break the symmetry in the end-to-end deep learning phase recovery.

In addition to expanding the application scenarios of this end-to-end deep learning strategy, some researchers focused on the performance and advantages of different neural networks in phase recovery. Xue et al.^132^ applied Bayesian neural network (BNN) into Fourier ptychography for inferring model uncertainty while doing phase recovery. Li et al.^133^ applied GAN for phase recovery, inferring the phase from two symmetric-illumination intensity images. Wang et al.^90,134^ proposed a one-to-multi CNN, Y-Net^90^, from which the amplitude and phase of an object can be inferred from the input intensity simultaneously. Zeng et al.^135^ introduce the capsule network to overcome information loss in the pooling operation and internal data representation of CNNs. Compared with conventional CNNs, their proposed capsule-based CNN (RedCap) saves 75% of network parameters while ensuring higher holographic reconstruction accuracy. Wu et al.^136^ applied the Y-Net^90^ to CDI for simultaneous inference of phase and amplitude. Huang et al.^137^ introduced a recurrent convolution module into U-Net, trained using GAN, for holographic reconstruction with autofocus. Uelwer et al.^138^ used a cascaded neural network for end-to-end phase recovery. Castaneda et al.^139^ and Jaferzadeh et al.^140^ introduced GAN into off-axis holographic reconstruction. Luo et al.^141^ added dilated convolutions into a CNN, termed mixed-context network (MCN)^141^, for phase recovery. By comparing in a one-sample-learning scheme, they found that MCN is more accurate and compact than the conventional U-Net. Ding et al.^142^ added Swin Transformer^143^ into U-Net and trained it with low-resolution intensity as input and high-resolution phase as ground truth using cycle-GAN. The trained neural network can do phase recovery while enhancing the resolution and has higher accuracy than the conventional U-Net. In CDI, Ye et al.^144^ used a multi-layer perceptron for feature extraction before a CNN, considering the property of the far-field (Fourier) intensity images where the data are globally correlated. Chen et al.^145,146^ combined the spatial Fourier transform module with ResNet, termed Fourier imager network (FIN), to achieve holographic reconstruction with superior generalization to new types of samples and faster inference speed (9-fold faster than their previous recurrent neural network, 27-fold faster than conventional iterative algorithms). Shu et al.^147^ applied neural architecture search (NAS) to automatically optimize the network architecture for phase recovery. Compared with the conventional U-Net, the peak signal-to-noise ratio (PSNR) of their NAS-based network is increased from 34.7 dB to 36.1 dB, and the inference speed is increased by 27-fold.

As a similar deep learning phase recovery strategy in adaptive optics, researchers demonstrated that neural networks could be used to infer the phase of the turbulence-induced aberration wavefront or its Zernike coefficient from the distortion intensity of target objects^148^. In these applications, only the wavefront subsequently used for aberration correction is of interest, not the RI distribution of turbulence that produces this aberration wavefront.

#### Physics-driven approach

**Fig. 18:**
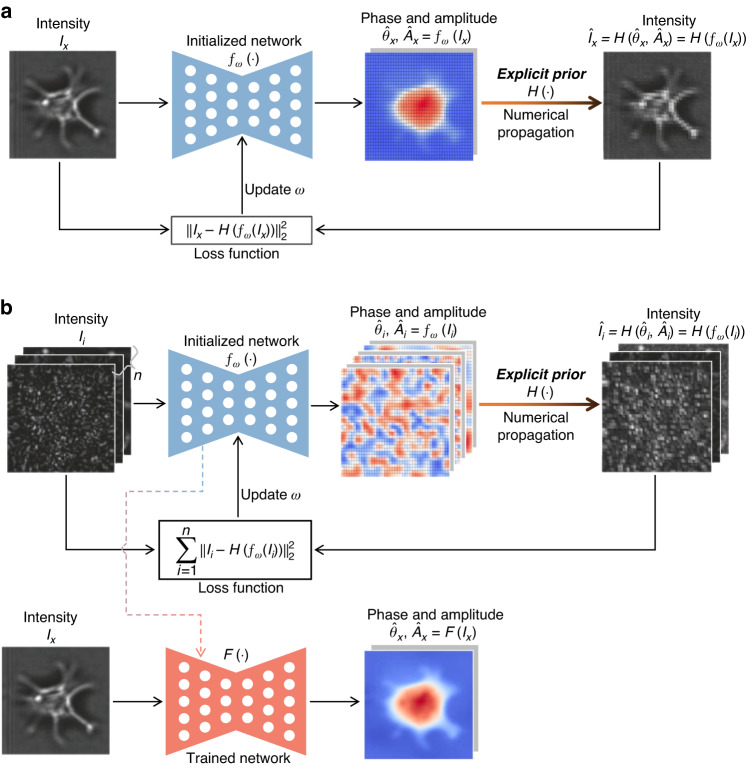
Description of physics-driven network-only phase recovery. **a** Untrained PD (uPD) scheme. **b** Trained PD (tPD) scheme

Different from the dataset-driven approach that uses input-label paired dataset as an *implicit prior* for neural network training, physical models, such as numerical propagation, can be used as an *explicit prior* to guide/drive the inference or training of neural networks, termed physics-driven (PD) approach. It only requires measurements of samples as an input-only dataset and is therefore an unsupervised learning mode. On the one hand, this *explicit prior* can be used to iteratively optimize an untrained neural network to infer the corresponding phase and amplitude from the measured intensity image as input, referred to as the untrained PD (uPD) scheme (Fig. 18a). On the other hand, this *explicit prior* can be used to train an untrained neural network with a large number of intensity images as input, which then can infer the corresponding phase from unseen intensity images, an approach called the trained PD (tPD) scheme (Fig. 18b).

In order to more intuitively understand the difference and connection between the DD and PD approaches, let us compare the loss functions in Fig. 17 and Fig. 18:

1 $$Los{s}_{DD}=\mathop{\sum }\limits_{i=1}^{n}{\Vert {f}_{\omega }({I}_{i})-{\theta }_{i}\Vert }_{2}^{2}$$

2 $$Los{s}_{uPD}={\Vert {I}_{x}-H({f}_{\omega }({I}_{x}))\Vert }_{2}^{2}$$

3 $$Los{s}_{tPD}=\mathop{\sum }\limits_{i=1}^{n}{\Vert {I}_{i}-H({f}_{\omega }({I}_{i}))\Vert }_{2}^{2}$$

where ${\Vert \cdot \Vert }_{2}^{2}$ denotes the square of the *l*_2_-norm (or other distance functions), ${f}_{\omega }(\cdot )$ is a neural network with trainable parameters $\omega$, $H(\cdot )$ is a physical model (such as numerical propagation, Fourier transform, or Fourier ptychography measurement model), ${I}_{i}$ is the measured intensity image in the training dataset, ${\theta }_{i}$ is the phase in the training dataset, ${I}_{x}$ is the measured intensity image of a test sample, and $n$ is the number of samples in the training dataset. In Eq. (1) for the DD approach, the priors used for network training are the measured intensity image and corresponding ground-truth phase. Meanwhile, in Eqs. (2) and (3) for the PD approaches, the priors used for network inference or training are the measured intensity image and physical model, instead of the phase. It should be noted that *the uPD scheme is free from numerous intensity images as a prerequisite, but requires numerous iterations for each inference; while the tPD scheme completes the inference only passing through the trained neural network once, but requires a large number of intensity images for pretraining*.

This PD approach was first implemented in the work on Fourier ptychography by Boominathan et al.^149^. They proposed it in the higher overlap case, including the scheme of directly using an untrained neural network for inference (uPD) and the scheme of training first and then inferring (tPD), and demonstrated the former by simulation.

For the uPD scheme, Wang et al.^150^ used a U-Net-based scheme to iteratively infer the phase of a phase-only object from a measured diffraction image whose de-focus distance is known. Their method demonstrates higher accuracy than conventional algorithms (such as GS and TIE) and the DD scheme, at the expense of a longer inference time (about 10 minutes for an input with 256 × 256 pixels). Zhang et al.^151^ extended this work to the case where the defocus distance is unknown by including it as another unknown parameter together with the phase to the loss function. Yang et al.^152,153^ found that after expanding the tested sample from phase-only to complex-amplitude, obvious artifacts and noise appeared in the recovered results. Therefore, they proposed to add an aperture constraint into the loss function to reduce the ill-posedness of the problem. Regarding the timeliness, they pointed out that it would cost as much as 600 hours to infer 3,600 diffraction images with this uPD scheme. Meanwhile, Bai et al.^154^ extended this from a single-wavelength case to a dual-wavelength case. Galande et al.^155^ found that this way of neural network optimization with a single-measurement intensity input lacks information diversity and can easily lead to overfitting of the noise, which can be mitigated by introducing an explicit denoiser. It is worth pointing out that *this way of using the object-related intensity image as the neural network input makes it possible to internalize the mapping relationship between intensity and phase into the neural network through pre-training*. In addition, some researchers proposed to make adjustments to the uPD scheme, using the initial phase and amplitude recovered by backward numerical propagation as the neural network input^156–158^, which reduces the burden on the neural network to obtain higher inference accuracy.

Although the phase can be inferred from the measured intensity image through an untrained neural network without any ground truth, the uPD scheme inevitably requires a large number of iterations, which excludes its use in many dynamic applications. Therefore, to adapt the PD scheme to dynamic inference, Yang et al.^152,153^ adjusted their previously proposed uPD scheme to the tPD scheme by pre-training the neural network using a small part of the measured diffraction images, and then using the pre-trained neural network to infer the remaining ones. Yao et al.^159^ trained a 3D version of the Y-Net^90^ with simulated diffraction images as input, and then used the pre-trained neural network for direct inference or iterative refinement, which is 100 and 10 times faster than conventional iterative algorithms, respectively. Li et al.^160^ proposed a two-to-one neural network to reconstruct the complex field from two axially displaced diffraction images. They used 500 simulated diffraction images to pre-train the neural network, and then inferred an unseen diffraction image by refining the pre-trained neural network for 100 iterations. Bouchama et al.^161^ further extended the tPD scheme to Fourier ptychography of low overlap cases by simulated datasets. Different from the above ways of generating training datasets from natural images or real experiments, Huang et al.^162^ proposed to generate holograms as training datasets from randomly synthesized artificial images with no connection or resemblance to real-world samples. They further trained a neural network with the generated holograms and the tPD scheme, which showed superior external generalization to holograms of real tissues with arbitrarily defocus distances. It is worth mentioning that the PD strategy can also be used in computer-generated holography, generating the corresponding hologram from the target phase or amplitude via a physics-driven neural network^163,164^.

### Network-with-physics strategy

Different from the network-only strategy, in the network-with-physics strategy, either the physical model and neural network are connected in series for phase recovery (physics-connect-network, PcN), or the neural network is integrated into a physics-based algorithm for phase recovery (network-in-physics, NiP), or the physical model or physics-based algorithm is integrated into a neural network for phase recovery (physics-in-network, PiN). A summary of the network-with-physics strategy is presented in Table 3 and is described below.

**Table 3:** Summary of network-with-physics strategy

Task	Reference	Input	Output	Network	Training dataset	Loss function
Physics-connect-network (PcN)	Rivenson et al.^165^	Initial complex field	Pure complex field	CNN and ResNet	Expt.: 100 pairs	*l*_2_-norm
	Wu et al.^166^	Initial complex field	Pure complex field (in-focus)	U-Net and ResNet	Expt.: 704 pairs	*l*_1_-norm
	Huang et al.^137^	Initial complex field	Pure complex field	U-Net and Recurrent CNN	Expt.: 208 groups	GAN loss and *l*_1_-norm and SSIM
	Goy et al.^118^	Initial phase	Pure phase	U-Net and ResNet	Expt.: 9500 pairs	NPCC
	Deng et al.^168^	Initial phase	Pure phase	U-Net and ResNet	Expt.: 9500 pairs	*l*_2_-norm and features of VGG
	Deng et al.^169^	(i) Initial phase, (ii) LR and HR phase	(i) LR or HR phase, (ii) Phase	U-Net and ResNet (three)	Expt.: 9500 pairs	NPCC
	Kang et al.^170^	Initial phase	Pure phase	U-Net and ResNet	Expt.: 5000 pairs	NPCC or SSIM
	Zhang et al.^171^	Synthetic initial phase and amplitude	HR phase and amplitude	CNN and ResNet	Sim.: 23,040 groups	*l*_1_-norm
	Moon et al.^172^	initial superimposed phase	Pure phase	U-Net	Expt.: 1500 pairs	GAN loss
Network-in-physics (NiP)	Metzler et al.^174^	Noisy phase	Denoised phase	DnCNN	Sim.: 300,000 pairs	---
	Wu et al.^176^	Noisy phase	Denoised phase	DnCNN	Sim.: ---	---
	Bai et al.^177^	Noisy phase	Denoised phase	DnCNN	Sim.: 300,000 pairs	---
	Wang et al.^178^	Noisy phase	Denoised phase	DnCNN	Sim.: ---	*l*_2_-norm
	Chang et al.^179^	Noisy phase and amplitude	Denoised phase and amplitude	FFDNet	Sim.: 10,000 pairs	---
	Işıl et al.^180^	Noisy phase	Denoised phase	U-Net	Sim.: 3000 pairs	*l*_2_-norm
	Kumar et al.^181^	Noisy phase	Denoised phase	U-Net and ResNet	---	---
	Jagatap et al.^184,185^	Fixed vector	Phase	Decoder	Sim.: 1	*l*_2_-norm
	Zhou et al.^186^	Fixed matrix	Phase	SegNet	Sim. and Expt.: 1	*l*_2_-norm
	Shamshad et al.^187^	Fixed matrix	Phase	U-Net	Sim.: 1	*l*_2_-norm
	Bostan et al.^188^	Fixed vector and Zernike polynomials	Phase and aberrations	Decoder or fully connected network	Expt.: 1	*l*_2_-norm
	Lawrence et al.^189^	Fixed vector	Phase	Decoder	Sim.: 1	Poisson likelihood
	Niknam et al.^190^	Fixed vector	Phase and amplitude	Decoder	Expt.: 1	*l*_2_-norm
	Ma et al.^191^	Fixed vector	Phase	Decoder	Sim.: 1	*l*_2_-norm
	Chen et al.^192^	Fixed vector	Phase, amplitude, pupil aberration and illumination fluctuation factor	Decoders or fully connected networks	Sim.: 1	*l*_2_-norm
	Hand et al.^193^	Phase or random vector	Phase	VAE or DCGAN	Sim.: 60,000 and 200,000 pairs	*l*_2_-norm
	Shamshad et al.^194,195,197^	Random vector	Phase	DCGAN	Sim.: 60,000 73,257 pairs	*l*_2_-norm
	Hyder et al.^196^	Random vector	Phase	DCGAN	Sim.: 202,599 pairs	*l*_2_-norm
	Uelwer et al.^198^	Random vector	Phase	VAE, DCGAN or Style-GAN	Sim.: ---	*l*_2_-norm, LPIPS, and Wasserstein adversarial loss
Physics-in-network (PiN)	Wang et al.^200^	Intensity	Phase	deGEC-SR-Net	Sim.: 100 pairs	*l*_2_-norm
	Naimipour et al.^201,202^	Intensity	Phase	Auto-encoder network	Sim.: 2048 pairs	*l*_2_-norm
	Zhang et al.^203^	Intensity	Phase and amplitude	Complex U-Net (untrained)	Sim.: 1	*l*_2_-norm
	Shi et al.^204^	Intensity	Phase	Deep shrinkage network (DSN)	Sim.: 204,800 pairs	*l*_2_-norm
	Wu et al.^205^	Intensity	Phase	Cascaded CNN	Sim. and Expt.: 400 and 140 pairs	*l*_2_-norm
	Yang et al.^206^	Intensity	Phase	CNN in space and frequency domain	Sim.: 400 and 60,000 pairs	*l*_2_-norm and edge loss

“---” indicates not available

#### Physics-connect-network (PcN)

**Fig. 19:**
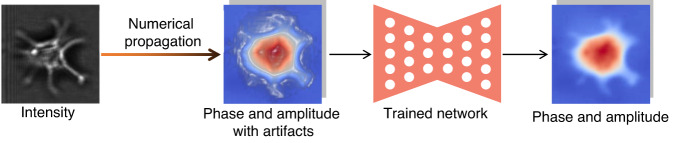
Description of physics-connect-network phase recovery

In this scheme, the role of the neural network is to extract and separate the pure phase from the initial estimate that may suffer from spatial artifacts or low resolution, which allows the neural network to perform a simpler task than the network-only strategy; typically, the initial phase is calculated using a physical model (Fig. 19). This scheme requires paired input-label datasets to teach the neural network and therefore belongs to supervised learning.

Rivenson et al.^165^ first applied this PcN scheme in holographic reconstruction in 2018. They used numerical propagation to calculate the initial complex field (including real and imaginary parts) from a single intensity-only hologram, which contained twin-image and self-interference-related spatial artifacts, and then used a data-driven trained neural network to extract the pure complex field from the initial estimate. Compared with the axial multi-intensity alternating projection algorithm^17–19^, their PcN scheme reduces the number of required holograms by 2–3 times while improving the computation time by more than three times. Wu et al.^166^ then extended the depth of field (DOF) based on this work by training a neural network with pairs of randomly de-focused complex fields and the corresponding in-focus complex field. Meanwhile, Huang et al.^137^ proposed the use of a recurrent CNN^167^ for the PcN scheme and the network-only strategy. They compared the performance of neural networks using either a hologram or an initial complex field as input within the same background and discovered that the network-only strategy is more robust for sparse samples, while the PcN scheme demonstrates better inference capabilities on dense samples. Goy et al.^118^ applied the PcN scheme to phase recovery under weak-light illumination, which is more ill-posed than conventional phase recovery. They showed that the inference performance of the PcN scheme is stronger than that of the network-only strategy under weak-light illumination, especially for dense samples in the extreme photon level case (1 photon). Further, Deng et al.^168^ introduced a default feature perceptual loss of the VGG layer into the loss function for neural network training, which inferred more fine details than that of the NPCC loss function. They also improved the spatial resolution and noise robustness by learning the low-frequency and high-frequency bands, respectively, through two neural networks and synthesizing these two bands into full-band reconstructions with a third neural network^169^. By introducing random phase modulation, Kang et al.^170^ further improved the phase recovery ability of the PcN scheme under weak-light illumination. Zhang et al.^171^ extended the PcN scheme to Fourier ptychography, inferring high-resolution phase and amplitude using the initial phase and amplitude synthesized from the intensity images as input to a neural network. Moon et al.^172^ extended the PcN scheme to off-axis holography, using numerical propagation to obtain the initial phase from the Gaber hologram as the input to the neural network.

#### Network-in-physics (NiP)

In this scheme, trained or untrained neural networks are used in physics-based iterative algorithms as denoisers, structural priors, or generative priors. Regarding phase recovery as one of the most general optimization problems, this approach can be expressed as

4 $${{\arg }}\mathop{\min }\limits_{\theta }{\Vert {I}_{x}-H(\theta )\Vert }_{2}^{2}+R(\theta )$$

where $H(\cdot )$ is the physical model, $\theta$ is the phase, ${I}_{x}$ is the measured intensity image of a test sample, and $R(\theta )$ is a regularized constraint. According to the Regularization-by-Denoising (RED)^173^ framework, a pre-trained neural network for denoising can be used as the regularized constraint:

5 $${{\arg }}\mathop{\min }\limits_{\theta }{\Vert {I}_{x}-H(\theta )\Vert }_{2}^{2}+\lambda {\theta }^{T}[\theta -D(\theta )]$$

where $D(\theta )$ is a pre-trained neural network for denoising, and $\lambda$ is a weight factor to control the strength of regularization. Metzler et al.^174^ used the above algorithm for phase recovery and called it PrDeep. They used a DnCNN trained on 300,000 pairs of data as a denoiser and FASTA^175^ as a solver. In comparison with other conventional iterative methods, PrDeep demonstrates excellent robustness to noise. Wu et al.^176^ proposed an online extension of PrDeep, which adopts the online processing of data by using only a random subset of measurements at a time. Bai et al.^177^ extended PrDeep to incorporate a contrast-transfer-function-based forward operator in $H(\cdot )$ for phase recovery. Wang et al.^178^ improved PrDeep by changing the solver from FASTA to ADMM, which further improved the noise robustness. Chang et al.^179^ used a generalized-alternating-projection solver to further expand the performance of PrDeep and made it suitable for the recovery of complex fields. Işıl et al.^180^ embedded a trained neural network denoiser into HIO, removing artifacts from the results after each iteration. On this basis, Kumar et al.^181^ added total-variation prior together with the denoiser for regularization.

**Fig. 20:**
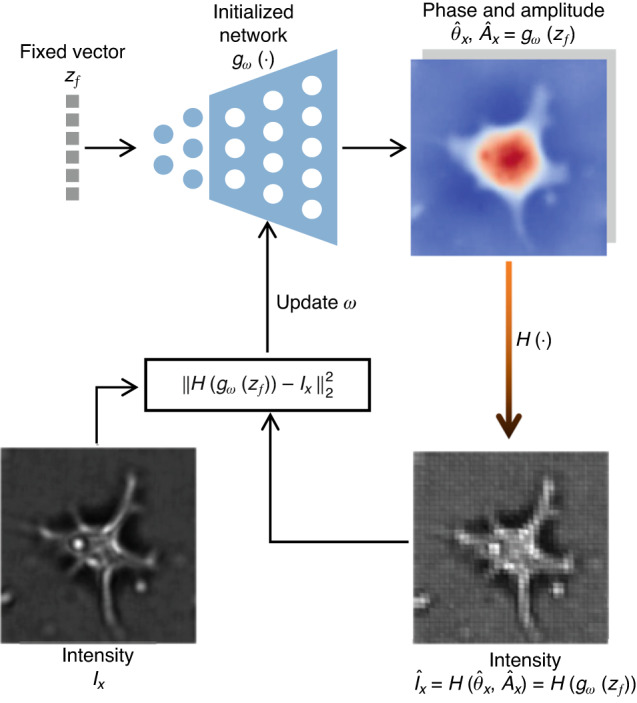
Description of structural-prior network-in-physics phase recovery

In addition, according to the deep image prior (DIP)^182,183^, even an untrained neural network itself can be used as a structural prior for regularization (Fig. 20):

6 $${{\arg }}\mathop{\min }\limits_{\omega }{\Vert {I}_{x}-H({g}_{\omega }({z}_{f}))\Vert }_{2}^{2}$$

where ${g}_{\omega }(\cdot )$ is an untrained neural network with trainable parameters $\omega$ that usually takes a generative decoder architecture, ${I}_{x}$ is the measured intensity image of a test sample, and ${z}_{f}$ is a fixed vector, which means that *the input of the neural network is independent of the sample, and therefore the neural network cannot be pre-trained like the PD approach*.

This DIP-based approach was first introduced to phase recovery by Jagatap et al.^184^. They solved Eq. (6) using the gradient descent and projected gradient descent algorithms by optimizing over trainable parameters $\omega$, both of which outperform sparse truncated amplitude flow (SPARTA) algorithm. In follow-up work, they provided rigorous theoretical guarantees for the convergence of their algorithm^185^. Zhou et al.^186^ applied this DIP-based algorithm to ODT, alleviating the effects of the missing cone problem. Shamshad et al.^187^ extended this DIP-based algorithm to subsampled Fourier ptychography, achieving better reconstructions at low subsampling ratios and high noise perturbations. In order to make the algorithm adaptive to different aberrations, Bostan et al.^188^ added a fully connected neural network with Zernike polynomials as the fixed input, and used it as the second structural prior. In the holographic setting with a reference beam, Lawrence et al.^189^ demonstrated the powerful information reconstruction ability of the DIP-based algorithm in extreme cases such as low photon counts, beamstop-obscured frequencies, and small oversampling. Niknam et al.^190^ used the DIP-based algorithm to recover complex fields from an in-line hologram. They further improved the twin-image artifacts suppression capability through some additional regularization, such as bounded activation function, weight decay, and parameter perturbation. Ma et al.^191^ embed an untrained generation network into the ADMM algorithm to solve the phase recovery at low subsampling ratios, and achieved better results than the gradient descent and projected gradient descent algorithms of Jagatap et al.^184^. Chen et al.^192^ extended the DIP-based algorithm to Fourier ptychography, in which four parallel untrained neural networks were used for generating phase, amplitude, pupil aberration, and illumination fluctuation factor correction, respectively.

**Fig. 21:**
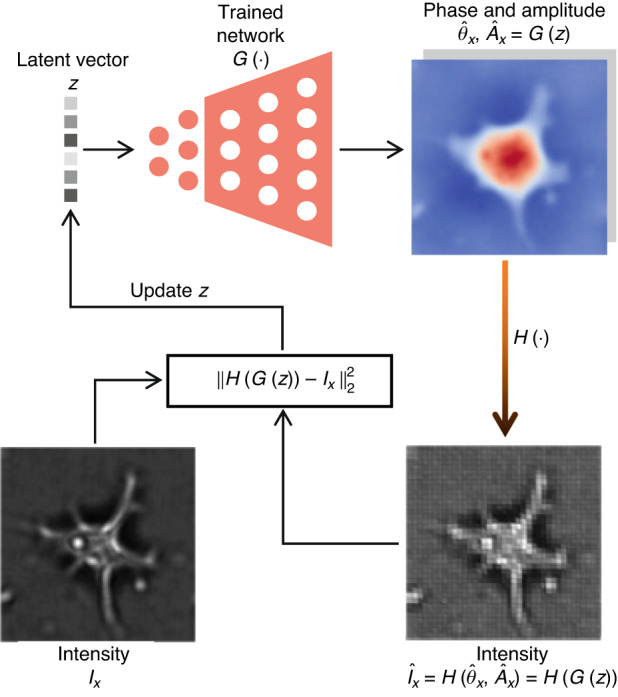
Description of generative-prior network-in-physics phase recovery

Similarly, a pre-trained generative neural network can also be used as a generative prior, assuming that the target phase is in the range of the output of this trained neural network (Fig. 21):

7 $${{\arg }}\mathop{\min }\limits_{z}{\Vert {I}_{x}-H(G(z))\Vert }_{2}^{2}$$

where $G(\cdot )$ is a pre-trained fixed neural network that usually takes a generative decoder architecture, ${I}_{x}$ is the measured intensity image of a test sample, and $z$ is a latent vector to be searched. Due to the use of the generative neural network, the multi-dimensional phase that originally needed to be iteratively searched is converted into a low-dimensional vector, and the solution space is also limited within the range of the trained generative neural network.

Hand et al.^193^ used the generative prior for phase recovery with rigorous theoretical guarantees for random Gaussian measurement matrix, showing better performance than SPARTA at low subsampling ratios. Later on, Shamshad et al.^194^ experimentally verified the robustness of the generative-prior-based algorithm to low subsampling ratios and strong noise in the coded diffraction setup. Then, Shamshad et al.^195^ extended this generative-prior-based algorithm to subsampled Fourier ptychography. Hyder et al.^196^ improved this by combining the gradient descent and projected gradient descent methods with AltMin-based non-convex optimization methods. As a general defect, the trained generative neural network will limit the solution space to a specific range related to the training dataset, so that the iterative algorithm cannot search beyond this range. Therefore, Shamshad et al.^197^ set both the input and previously fixed parameters of the trained generative neural network to be trainable. As another solution, Uelwer et al.^198^ extended the range of the trained generative neural network by intermediate layer optimization.

#### Physics-in-network (PiN)

**Fig. 22:**
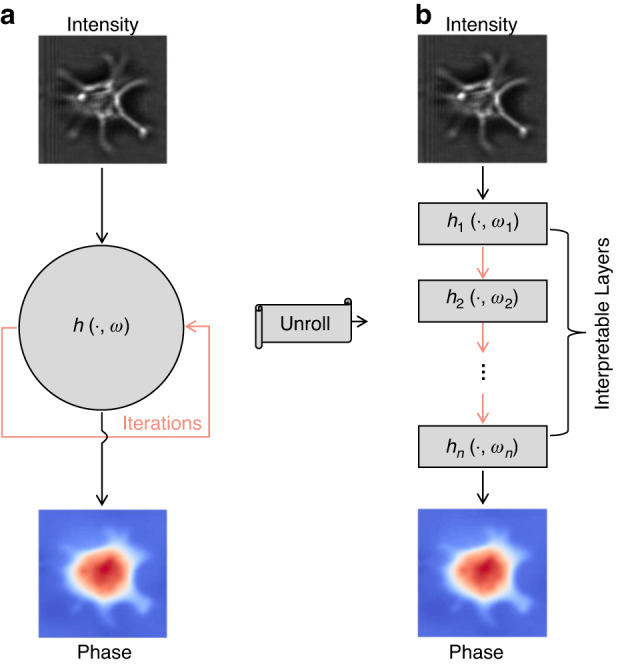
Description of physics-in-network phase recovery. **a** A physics-based iterative algorithm. **b** A corresponding unrolled neural network. The iteration step $h$ with algorithm parameters $\omega$ in (**a**) is unrolled and transferred to the network layers ${h}_{1}$, ${h}_{2}$,…, ${h}_{n}$ with network parameters ${\omega }_{1}$, ${\omega }_{2}$,…, ${\omega }_{n}$ in (**b**). The unrolled neural network is trained with the dataset in an end-to-end manner

According to the algorithm unrolling/unfolding technique proposed by Gregor and LeCun^199^, physics-based iterative algorithms can be unrolled as an interpretable neural network architecture (Fig. 22). Although this scheme integrates physics prior knowledge into neural networks, it still requires input-label paired datasets for neural network training and thus falls under the category of supervised learning. Wang et al.^200^ unrolled an algorithm called decentralized generalized expectation consistent signal recovery (deGEC-SR) into a neural network with trainable parameters, which exhibits stronger robustness using fewer iterations than the original deGEC-SR. Naimipour et al.^201,202^ used the algorithm unrolling technique in reshaped Wirtinger flow and SPARTA. Zhang et al.^203^ unrolled the iterative process of the alternative projection algorithm into complex U-Nets. Shi et al.^204^ used a deep shrinkage network and dual frames to unroll the proximal gradient algorithm in coded diffraction imaging. Wu et al.^205^ integrated the Fresnel forward operator and TIE inverse model into a neural network, which can be efficiently trained with a small number of datasets and is suitable for transfer learning. Yang et al.^206^ unrolled the classic HIO algorithm into a neural network that combines information both in the spatial domain and frequency domain. Since PiN-based networks are embedded with physical knowledge, good performance can usually be achieved with a small training dataset. It is worth mentioning that, as another type of PiN scheme, physics-informed neural networks mainly solves partial differential equations by embedding initial conditions, boundary conditions, and equation constraints into the loss function of neural networks^207^.

### Summary of “DL-in-processing for phase recovery”

At the end of this section, we provide a summary of “DL-in-processing for phase recovery” in Table 4, where “supervised learning mode” requires paired datasets, “weak-supervised learning mode” requires unpaired datasets, and “unsupervised learning mode” requires input-only, phase-only, or no datasets.

**Table 4:** Summary of all strategies in “DL-in-processing for phase recovery”

Strategy	Network task	Input	Output	Dataset	Learning mode
Network-only by dataset-driven	Phase recovery	Hologram	Phase	Paired dataset	Supervised
				Unpaired dataset	Weak-supervised
Network-only by untrained physics-driven	Phase recovery	Hologram	Phase	No requirement	Unsupervised
Network-only by trained physics-driven	Phase recovery	Hologram	Phase	Input-only dataset	Unsupervised
Physics-connect-network	Artifacts removal	Initial phase	Phase	Paired dataset	Supervised
Network-in-physics with denoisers	Regularization	Noisy phase	Phase	Paired dataset	Supervised
Network-in-physics with structural priors	Regularization	Fixed vector	Phase	No requirement	Unsupervised
Network-in-physics with generative priors	Regularization	Latent vector	Phase	Phase-only dataset	Unsupervised
Physics-in-network with interpretability	Phase recovery	Hologram	Phase	Fewer paired dataset	Supervised

## DL-post-processing for phase recovery

A summary of “DL-post-processing for phase recovery” is presented in Table 5 and is described below, including the “Noise reduction”, “Resolution enhancement”, “Aberration correction”, and “Phase unwrapping” sections.

**Table 5:** Summary of “DL-post-preprocessing for phase recovery”

Task	Reference	Input	Output	Network	Training dataset	Loss function
Noise reduction	Jeon et al.^208^	Noisy hologram	Noise-free hologram	U-Net	Sim.: 384,000 pairs	*l*_2_-norm and Edge
	Choi et al.^209^	Noisy tomogram	Noise-free tomogram	U-Net	Expt.: 455 and 5,057 (unpaired)	Cycle-GAN loss
	Zhang et al.^210^	Noisy wrapped phase	Noise-free wrapped phase	CNN	Sim.: 500 pairs	*---*
	Yan et al.^211,212^	Noisy sine and cosine	Noise-free sine and cosine	ResNet	Sim.: 40,000 and 30,000 pairs	*l*_2_-norm
	Montresor et al.^213^	Noisy sine and cosine	Noise-free sine and cosine	DnCNN	Sim.: 40 pairs (15,360 patches)	*l*_2_-norm
	Tahon et al.^214,215^	Noisy sine and cosine	Noise-free sine and cosine	DnCNN	Sim.: 25 pairs and 128 pairs	*l*_2_-norm
	Fang et al.^216^	Noisy real, imaginary	Noise-free real, imaginary	U-Net	Sim.: 4000 pairs	GAN loss
	Murdaca et al.^217^	Noisy real, imaginary, and amplitude	Noise-free real, imaginary, and amplitude	U-Net	Sim.: 5400 pairs	*l*_2_-norm
	Tang et al.^219^	Fixed noise matrix	Noise-free phase	U-Net (untrained)	Expt.: 1	*l*_2_-norm, gradient, and variance
Resolution enhancement	Liu et al.^220^	LR phase and amplitude	HR phase and amplitude	U-Net	Expt.: >50,000 pairs	GAN loss
	Jiao et al.^221^	LR phase from DPM	HR phase from SLIM	U-Net	Expt.: >1200 pairs (>100 cells)	*l*_2_-norm
	Butola et al.^223^	LR phase	HR phase	U-Net	Expt.: 2355 pairs and 2279 pairs	GAN loss
	Meng et al.^224^	LR phase from SI-DHM	HR phase from SI-DHM	U-Net	Expt.: 3800 pairs	*l*_2_-norm
	Li et al.^226^	LR phase	HR phase	U-Net	Expt.: 1680 pairs	*l*_2_-norm
	Gupta et al.^227^	LR phase	HR phase	U-Net	Expt.: 700–2000 (unpaired)	Cycle-GAN loss
	Lim et al.^228^	LR 3D RI tomogram	HR 3D RI tomogram	Residual 3D U-Net	Sim.: 1600 pairs	*l*_2_-norm
	Ryu et al.^229^	LR 3D RI tomogram	HR 3D RI tomogram	3D U-Net	Expt.: 217 and 614 pairs	*l*_2_-norm
Aberration correction	Nguyen et al.^234^	Phase	Binary segmentation	U-Net	Expt.: 1836 pairs	Cross entropy
	Ma et al.^235^	Hologram	Binary segmentation	U-Net	Expt.: 1000 pairs	Cross entropy
	Lin et al.^236^	Phase and its gradient	Binary segmentation	U-Net and ResNet	Expt.: 1800 pairs	Dice loss
	Xiao et al.^237^	Phase	Zernike coefficient	CNN	Expt.: 10,000 pairs	*l*_2_-norm
	Zhang et al.^238^	Aberrated intensity and phase	Phase	U-Net	Sim.: >10,000 groups	*l*_2_-norm or *l*_1_-norm
	Tang et al.^239^	Fixed vector	Zernike coefficient	MLP (untrained)	Expt. and Sim.: 1	*l*_2_-norm and sparse constraints
Phase unwrapping	Dardikman et al.^243,244^	Wrapped phase	Unwrapped phase	ResNet	Sim.: 7936 pairs	*l*_2_-norm
	Wang et al.^245^	Wrapped phase	Unwrapped phase	U-Net and ResNet	Sim.: 30,000 pairs	*l*_2_-norm
	He et al.^246^	Wrapped phase	Unwrapped phase	3D-ResNet	Expt.: ---	*---*
	Ryu et al.^247^	Wrapped phase	Unwrapped phase	ReNet	Expt. and Sim.: ---	Total variation and variance
	Dardikman et al.^248^	Wrapped phase	Unwrapped phase	ResNet	Expt.: 7500 pairs	*l*_2_-norm
	Qin et al.^249^	Wrapped phase	Unwrapped phase	U-Net and ResNet	Sim.: 30,000 pairs	*L*_1_-norm
	Perera et al.^250^	Wrapped phase	Unwrapped phase	U-Net and LSTM	Sim.: 6000 pairs	Total variation and variance
	Park et al.^251^	Wrapped phase	Unwrapped phase	U-Net	Expt.: 5200 pairs	GAN loss
	Zhou et al.^252^	Wrapped phase and wrap count	Unwrapped phase	U-Net and EfficientNet	Sim.: 6000 pairs	*l*_1_-norm and residual
	Xu et al.^253^	Wrapped phase	Unwrapped phase	U-Net	Sim.: 6000 pairs	SSIM
	Zhou et al.^254^	Wrapped phase	Unwrapped phase	U-Net	Sim.: 158 and 1036 pairs	GAN loss
	Xie et al.^255^	Wrapped phase	Unwrapped phase	U-Net	Sim.: 17,000 pairs	*l*_2_-norm
	Zhao et al.^256^	Wrapped phase and weighted map	Unwrapped phase	U-Net and ResNet	Sim.: 22,500 pairs	*l*_1_-norm
	Liang et al.^257^	Wrapped phase	Wrap count	---	---	*---*
	Spoorthi et al.^258^	Wrapped phase	Wrap count	SegNet	Sim.: 10,000 pairs	Cross entropy
	Spoorthi et al.^259^	Wrapped phase	Wrap count	SegNet and DenseNet	Sim.: 30,000 pairs	Cross entropy and residue and *l*_1_-norm
	Zhang and Liang et al.^210,260^	Wrapped phase	Wrap count	U-Net	Sim.: 9500 pairs	Cross entropy
	Zhang et al.^261^	Wrapped phase	Wrap count	DeepLab-V3+	Sim.: 25,000 pairs	Cross entropy
	Zhu et al.^262^	Wrapped phase	Wrap count	DeepLab-V3+	Sim.: 20,000 pairs	Cross entropy
	Wu et al.^263^	Wrapped phase	Wrap count	U-Net and FRRNet	Sim.: 12,000 pairs	Cross entropy
	Zhao et al.^264^	Wrapped phase	Wrap count	ResNet	Sim.: 22,000 pairs	Cross entropy
	Vengala et al.^265,266^	Wrapped phase	Wrap count and denoised wrapped phase	Y-Net	Sim.: 2000 pairs	Cross entropy and *l*_2_-norm
	Zhang et al.^267^	Wrapped phase	Wrap count	U-Net and ASPP and PSA	Sim.: 10,000 pairs	Weighted cross entropy
	Huang et al.^278^	Wrapped phase	Wrap count	HRNet	Sim.: 30,000 pairs	Cross entropy
	Wang et al.^279^	Wrapped phase	Wrap count	U-Net, ASPP and EEB	Sim.: ---	Cross entropy
	Zhou et al.^270^	Wrapped count	Wrap count gradient	CNN	Sim.: 52,391 pairs	Cross entropy
	Wang et al.^271^	Wrapped count and quality map	Wrap count gradient	U-Net	Sim.: 164,726 pairs	Cross entropy and dice loss
	Sica et al.^268^	Wrapped count	Wrap count gradient	U-Net	Sim.: >70,000 pairs	Cross entropy, Jaccard distance, and *l*_1_-norm
	Li et al.^269^	Wrapped count	Wrap count gradient	U-Net and ResNet	Sim.: 14,100 pairs	Cross entropy
	Wu et al.^272,273^	Wrapped count	Discontinuity map	CNN and ASPP	Sim.: 8000 pairs	*l*_2_-norm, cross entropy, and dice loss
	Zhou et al.^274^	Residue image	Branch-cut map	CNN	Sim.: 26,928 pairs	Cross entropy

“---” indicates not available

### Noise reduction

**Fig. 23:**
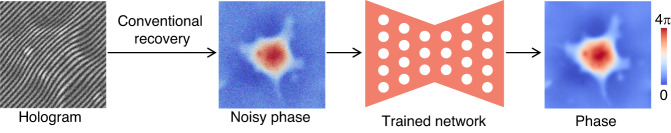
Description of deep-learning-based phase noise reduction

In addition to being part of the pre-processing in “Noise reduction” under the section “DL-pre-processing for phase recovery”, noise reduction can also be performed after phase recovery (Fig. 23). Jeon et al.^208^ applied the U-Net to perform speckle noise reduction on digital holographic images in an end-to-end manner. Their deep learning method takes only 0.92 s for a reconstructed hologram of 2048 × 2048, while other conventional methods take tens of seconds because of the requirement of multiple holograms. Choi et al.^209^ introduced the cycle-GAN to train neural networks for noise reduction by unpaired datasets. They demonstrated the advantages of this un-paired-data-driven method with tomograms of different cell samples in optical diffraction chromatography: the non-data-driven ways either remove coherent noise by blurring the entire images or perform no effective denoising, whereas their method can simultaneously remove the noise and preserve the features of the sample.

Zhang et al.^210^ first proposed to suppress noise directly on the wrapped phase via a neural network. However, this direct way may lead to many wrong jumps in the wrapped phase, which results in larger errors in the unwrapped phase. Thus, Yan et al.^211,212^ proposed to do noise reduction on the sine and cosine (numerator and denominator) images of the phase via a neural network, and then calculated the wrapped phase from denoised sine and cosine images by the arctangent function. Almost simultaneously, Montresor et al.^213^ introduced the DnCNN into speckle noise reduction for phase data by their sine and cosine images. As it is difficult to simultaneously collect the phase data with and without speckle noise in an experimental manner, they used a simulator based on a double-diffraction system to numerically generate the dataset. Furthermore, their method yields comparable standard deviation to the WFT and better peak-to-valley, while costing less time. Building on this work, Tahon et al.^214^ designed a dataset (HOLODEEP) for speckle noise reduction in soft conditions and used a shallower network for faster inference. To go further, they released a more comprehensive dataset for conditions of severe speckle noise^215^. Fang et al.^216^ applied GAN to do speckle noise reduction for phase. Murdaca et al.^217^ applied this deep-learning-based phase noise reduction to interferometric synthetic aperture radar (InSAR)^218^. The difference is that in addition to the sine and cosine images of the phase, the neural network also reduces noise for the amplitude images at the same time. Tang et al.^219^ proposed to iteratively reduce the coherent noise in phase with an untrained U-Net. In the above works, various loss functions were employed alongside the conventional *l*_2_-norm and *l*_1_-norm to enhance performance. These additional functions include the edge function^208^, which sharpens the edges of the denoised image, as well as gradient and variance functions^219^ that further suppress noise while preventing excessive smoothing.

### Resolution enhancement

**Fig. 24:**
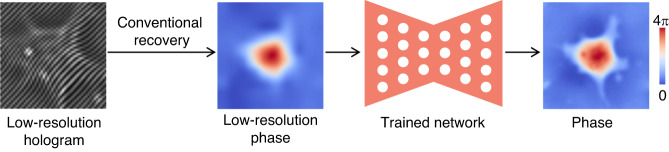
Description of deep-learning-based phase resolution enhancement

Similar to the section “Pixel super-resolution”, resolution enhancement can also be performed after phase recovery as post-processing (Fig. 24). Liu et al.^220^ first used a neural network to infer the corresponding high-resolution phase from the low-resolution phase. They trained two GANs with both a pixel super-resolution system and a diffraction-limited super-resolution system, which was demonstrated on biological thin tissue slices with the analysis of spatial frequency spectrum. Moreover, they pointed out that this idea can be extended to other resolution-limited imaging systems, such as using a neural network to build a passageway from off-axis holography to in-line holography. Later, Jiao et al.^221^ proposed to infer the high-resolution noise-free phase from an off-axis-system-acquired low-resolution version with a trained U-Net. To collect the paired dataset, they developed a combined system with diffraction phase microscopy (DPM)^222^ and spatial light interference microscopy (SLIM)^27^ to generate both holograms from the same field of view. After training, the U-Net retains the advantages of both the high acquisition speed of DPM and the high transverse resolution of SLIM.

Subsequently, Butola et al.^223^ extended this idea to partially spatially coherent off-axis holography, where the phase recovered at low-numerical-apertures objectives was used as input, and the phase recovered at high-numerical-apertures objectives was used as ground truth. Since low-numerical-apertures objectives have a larger field of view, they aim to obtain a higher resolution at a larger field of view, i.e., a higher spatial bandwidth product. Meng et al.^224^ used structured-illumination digital holographic microscopy (SI-DHM)^225^ to collect the high-resolution phase as ground truth. To supplement more high-frequency information by two cascaded neural networks, they used the low-resolution phase along with the high-resolution amplitude inferred from the first neural network both as inputs of the second neural network. Subsequently, Li et al.^226^ extended this resolution-enhanced post-processing method to quantitative differential phase contrast microscopy for high-resolution phase recovery from the least number of experimental measurements. To solve the problem of out-of-memory for the large size of the input, they disassembled the full-size input into some sub-patches. Moreover, they found that the U-Net trained on the paired dataset has a smaller error than the paired GAN and the unpaired GAN. For GAN, there is more unreasonable information in the inferred phase, which is absent in ground truth. Gupta et al.^227^ took advantage of the high spatial bandwidth product of this method to achieve a classification throughput rate of 78,000 cells per second with an accuracy of 76.2%. All these works use U-Net as the basic structure, where most neural networks input and output phase maps of the same size and thus have the same number of downsampling times and upsampling times, whereas for the application where the input size is smaller than the output^227^, the neural network has more upsampling times.

For ODT, due to the limited projection angle imposed by the numerical aperture of the objective lens, there are certain spatial frequency components that cannot be measured, which is called the missing cone problem. To address this problem via a neural network, Lim et al.^228^ and Ryu et al.^229^ built a 3D RI tomogram dataset for 3D U-Net training, in which the raw RI tomograms with poor axial resolution were used as input, and the resolution-enhanced RI tomograms from the iterative total variation algorithm were used as ground truth. The trained 3D U-Net can infer the high-resolution version directly from the raw RI tomograms. They demonstrated the feasibility and generalizability using bacterial cells and a human leukemic cell line. Their deep-learning-based resolution-enhanced method outperforms conventional iterative methods by more than an order of magnitude in regularization performance.

### Aberration correction

For holography, especially in the off-axis case, the lens and the unstable environment of the sample introduce phase aberrations superimposing on the phase of the sample. To recover the pure phase of the sample, the unwanted phase aberrations should be eliminated physically or numerically. Physical approaches compensate for the phase aberrations by recovering the background phase without the sample from anther hologram, which requires more setups and adjustments^230,231^.

**Fig. 25:**
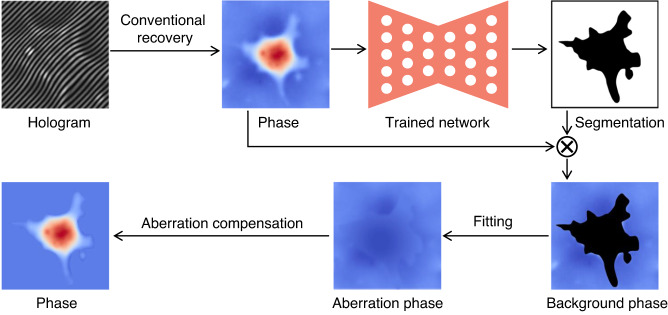
Description of deep-learning-based phase aberration correction

As for numerical approaches, the compensation of the phase aberrations can be directly achieved by Zernike polynomial fitting (ZPF)^232^ or principal-component analysis (PCA)^233^. Yet, in these numerical methods, the aberration is predicted from the whole phase, where the object area should not be considered as an aberration. Thus, before using the Zernike polynomial fitting, the neural network can be used to find out the object area and the background area to avoid the influence of the background area and improve the compensation effect (Fig. 25). This segmentation-based idea, namely CNN + ZPF, was first proposed by Nguyen et al.^234^ in 2017. They manually made binary masks as ground truth for each phase to distinguish the area of the background and sample. After comparison on different real samples, they found that the compensated result of the CNN + ZPF contains a flatter background than that of PCA. However, the aberration in the initial phase makes it more difficult to do segmentation from the already weak phase distribution of the boundary features, especially for the large tilted phase aberrations. To address this problem, Ma et al.^235^ proposed to do segmentation with hologram instead of phase as neural network input. Lin et al.^236^ applied the CNN + ZPF to real-time phase compensation with a phase-only SLM.

In addition to the way of CNN + ZPS, Xiao et al.^237^ directly inferred the Zernike coefficient of aberration from the initial phase via a neural network, which costs less computation. They trained a neural network specifically for bone cells, and used this efficient method to achieve long-term morphological observation of living cells. Zhang et al.^238^ used a trained neural network to infer the in-focus phase from the de-focus aberrated intensity and phase. Tang et al.^239^ introduced the sparse constraint into the loss function and iteratively inferred the corresponding phase aberrations from the initial phase or fixed vector with an untrained neural network and Zernike model.

### Phase unwrapping

**Fig. 26:**
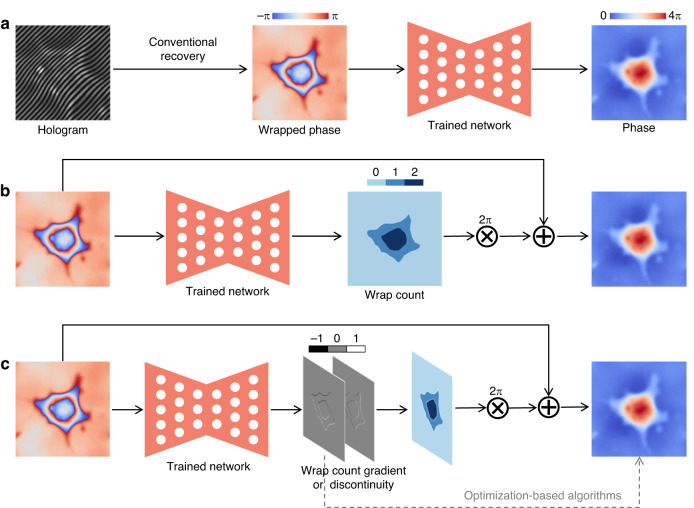
Description of deep-learning-based phase unwrapping. **a** Deep-learning-performed regression method. **b** Deep-learning-performed wrap count method. **c** Deep-learning-assisted method

In the interferometric and optimization-based phase recovery methods, the recovered light field is in the form of complex exponential. Hence, the calculated phase is limited in the range of (-π, π] on account of the arctangent function. Therefore, the information of the sample cannot be obtained unless the absolute phase is first estimated from the wrapped phase, the so-called phase unwrapping. In addition to phase recovery, the phase unwrapping problem also arises in magnetic resonance imaging^240^, fringe projection profilometry^241^, and InSAR. Most conventional methods are based on the phase continuity assumption, and some cases, such as noise, breakpoints, and aliasing, all violate the Itoh condition and affect the effect of the conventional methods^242^. The advent of deep learning has made it possible to perform phase unwrapping in the above cases. According to the different uses of the neural network, these deep-learning-based phase unwrapping methods can be divided into the following three categories (Fig. 26)^66^. Deep-learning-performed regression method (dRG) estimates the absolute phase directly from the wrapped phase by a neural network (Fig. 26a)^243–256^. Deep-learning-performed wrap count method (dWC) first estimates the wrap count from the wrapped phase by a neural network, and then calculates the absolute phase from the wrapped phase and the estimate wrap count (Fig. 26b)^210,257–267^. Deep-learning-assisted method (dAS) first estimates the wrap count gradient or discontinuity from the wrapped phase by a neural network; next, either reconstruct the wrap count from the wrap count gradient and then calculate the absolute phase like dWC^268,269^, or directly use optimization-based or branch-cut algorithms to obtain the absolute phase from the warp count gradient or the discontinuity (Fig. 26c)^270–274^.

#### Deep-learning-performed regression method (dRG)

Dardikman et al.^243^ presented the dRG method, which utilizes a residual-block-based CNN with a dataset of simulated steep cells. They also validated the dRG method post-processed by congruence in actual cells and compared it with the performance of the dWC method^244^. Then, Wang et al.^245^ introduced the U-Net and a phase simulation generation method into the dRG method, wherein they evaluated the trained network on real samples, examined the network’s generalization ability through middle-layer visualization, and demonstrated the superiority of the dRG method over conventional methods in noisy and aliasing cases. In the same year, He et al.^246^ and Ryu et al.^247^ evaluated the ability of the 3D-ResNet and recurrent neural network (ReNet) to perform phase unwrapping using magnetic resonance imaging data. Dardikman et al.^248^ released their real sample dataset as open-source. They demonstrated that the congruence could enhance the accuracy and robustness of the dRG method, particularly when dealing with a limited number of wrap count. Qin et al.^249^ utilized a Res-UNet with a larger capacity to achieve higher accuracy and introduced two new evaluation indices. Perera et al.^250^ and Park et al.^251^ introduced the long short-term memory (LSTM) network and GAN into phase unwrapping. Zhou et al.^252,275^ enhanced the robustness and efficiency of the dRG method by doing preprocessing and postprocessing steps for the U-Net with EfficientNet^275^ backbone. Xu et al.^253^ improved the accuracy and robustness of the U-Net by adding more middle-layers and skip connections and using a composite loss function. Zhou et al.^254^ used the GAN in the InSAR phase unwrapping and avoided the blur in the unwrapped phase by combining the *l*_1_ loss and adversarial loss. Xie et al.^255^ trained four networks for different noise levels, which made each network more focused on a specific noise level. Zhao et al.^256^ added a weighted map as the prior to the neural network to make it more focused on the area near the jump edge, similar to an additional attention mechanism. Different from the above methods, Vithin et al.^276,277^ proposed to use the Y-Net^90^ to infer the phase gradients from a wrapped phase and then calculate the absolute phase.

#### Deep-learning-performed wrap count method (dWC)

Liang et al.^257^ and Spoorthi et al.^258^ first proposed this idea in 2018. Spoorthi et al.^258^ proposed a phase dataset generation method by adding and subtracting Gaussian functions with randomly varying mean and variance values, and used the clustering-based smoothness to alleviate the classification imbalance of the SegNet. Further, the prediction accuracy of their methods was improved by introducing the prior of absolute phase values and gradients into the loss function, which they called Phase-Net2.0^259^. Zhang and Liang et al.^210,260^ sequentially used three networks to perform phase unwrapping by wrapped phase denoising, wrap count predicting, and post-processing. In addition, they proposed to generate a phase dataset by weighted adding Zernike polynomials of different orders. Immediately after, Zhang and Yan et al.^261^ verified the performance of the network DeepLab-V3+, but the resulting wrap count still contained a small number of wrong pixels, which will propagate error through the whole phase maps in the conventional phase unwrapping process. They thus proposed to use refinement to correct the wrong pixels. To further improve the unwrapped phase, Zhu et al.^262^ proposed to use the median filter for the second post-processing to correct wrong pixels in the wrap count predictions. Wu et al.^263^ enhanced the simulated phase dataset by adding the noise from real data. They also used the full-resolution residual network (FRRNet) with U-Net to further optimize the performance of the U-Net in the Doppler optical coherence tomography. By comparison with real data, their proposed network holds a higher accuracy than that of the Phase-Net and DeepLab-V3+. As for applying the dWC to point diffraction interferometer, Zhao et al.^264^ proposed an image-analysis-based post-processed method to alleviate the classification imbalance of the task and adopted the iterative-closest-point stitching method to realize dynamic resolution. Vengala et al.^90,265,266^ used the Y-Net^90^ to reconstruct the wrap count and pure wrapped phase at the same time. Zhang et al.^267^ added atrous spatial pyramid pooling (ASPP), positional self-attention (PSA), and edge-enhanced block (EEB) to the U-Net to get higher accuracy and stronger robustness than the networks used in the above methods. Huang et al.^278^ applied the HRNet to the dWC methods. Their method still needs the median filter for post-processing, although the performance is better than that of the Phase-Net and DeepLab-V3+. Wang et al.^279^ proposed another EEB based on Laplacian and Prewitt edge enhancement operators for the network, which further enhances classification accuracy and avoids the use of post-processing.

#### Deep-learning-assisted method (dAS)

The conventional methods estimate the wrap count gradient under the phase continuity assumption, which hence is disturbed by unfavorable factors such as noise. To get rid of it, Zhou et al.^270^ proposed to estimate the wrap count gradient via a neural network instead of conventional methods. Since the noisy wrapped phase and the corresponding correct wrap count gradient are used as training datasets, the trained neural network is able to estimate the correct wrap count gradient from the noisy wrapped phase without being limited by the phase continuity assumption. The correct result can be obtained by minimizing the difference between the unwrapped phase gradients and the network-output wrap count gradient. Further, Wang et al.^271^ proposed to input a quality map, as the prior, together with the wrapped phase into the neural network to improve the accuracy of the estimated wrap count gradient. Almost simultaneously, Sica et al.^268^ directly reconstructed the wrap count from the network-output wrap count gradient and then calculated the absolute phase, like dWC. On this basis, Li et al.^269^ improved neural network estimation efficiency by using a single fusion gradient instead of the vertical and horizontal gradients. In addition to estimating the wrap count gradient via a neural network, Wu et al.^272,273^ chose to estimate the horizontal and vertical discontinuities with a neural network, and recover the absolute phase by the optimization-based algorithms. Instead of using the wrapped phase as the network input, Zhou et al.^274^ embedded the neural network into the branch-cut algorithm to predict the branch-cut map from the residual image, which reduced the computational cost of the branch-cut algorithm.

## Deep learning for phase processing

A summary of “Deep learning for phase processing” is presented in Table 6 and is described below, including the “Segmentation”, “Classification”, and “Imaging modal transformation” sections.

**Table 6:** Summary of “Deep learning for phase processing”

Task	Reference	Input	Output	Network	Training dataset	Loss function
Segmentation	Yi et al.^282^	Phase of red blood cells	Segmentation map	FCN	Expt.: 35 pairs	*---*
	Ahmadzadeh et al.^284^	Phase of cardiomyocyte	Segmentation map	FCN	Expt.: 2000 pairs	---
	Kandel et al.^285^	Phase of sperm cells	Segmentation map	U-Net	Expt.: ---	Cross entropy
	Goswami et al.^286^	Phase of virus particles	Segmentation map	U-Net	Expt.: 1000 pairs	Cross entropy
	Hu et al.^287^	Phase of ovary cells	Segmentation map	U-Net and EfficientNet	Expt.: 1536 pairs	Focal loss and dice loss
	He et al.^288^	Phase of HeLa cells	Segmentation map	U-Net and EfficientNet	Expt.: 2046 pairs	focal loss and dice loss
	Zhang et al.^289^	Phase of tissue slices	Segmentation map	mask R-CNN	Expt.: 196 pairs	Cross entropy
	Jiang et al.^290^	Phase and amplitude	Segmentation map	DeepLab-V3+	Expt.: 1500 pairs	Cross entropy
	Lee et al.^291^	2D RI tomogram	Segmentation map	U-Net	Expt.: 934 pairs	Cross entropy
	Choi et al.^292^	3D RI tomogram	Segmentation map	3D U-Net	Expt.: 105 pairs	Cross entropy and dice loss
Classification	Jo et al.^293^	Phase of cells	Classification	CNN	Expt.: ---	Cross entropy
	Karandikar et al.^314^	Phase of cells	Classification	CNN	Expt.: 300	Cross entropy
	Zhang et al.^315^	Phase of tissue slices	Classification	VGG	Expt.: 1660	Cross entropy
	Butola et al.^316^	Phase of sperm cells	Classification	CNN	Expt.: 10,163	Cross entropy
	Li et al.^317^	Phase of cells	Classification	AlexNet	Expt.: 272	Cross entropy
	Shu et al.^318^	Phase of cells	Classification	Cascaded ResNet	Expt.: 1521	Cross entropy
	Pitkäaho et al.^319^	Phase and manual feature	Classification	CNN	Expt.: 2451	---
	O’Connor et al.^320^	Transfer-learning and manual feature from phase	Classification	LSTM	Expt.: 303	---
	O’Connor et al.^321^	Transfer-learning and manual feature from phase	Classification for COVID-19	LSTM	Expt.: 1474	---
	Ryu et al.^322^	3D RI tomogram	Classification (2 and 5 types)	3D CNN	Expt.: 1782	Cross entropy
	Kim et al.^323^	3D RI tomogram	Classification (19 types)	3D CNN	Expt.: 10,556	Cross entropy
	Wang et al.^324^	Time-lapse amplitude and phase	Classification (3 types)	Pseudo-3D DensNet	Expt.: 16,309	Cross entropy
	Liu et al.^325^	Time-lapse phase	Classification	Pseudo-3D DensNet	Expt.: 5622	Cross entropy
	Ben Baruch et al.^326^	Phase and spatio-temporal fluctuation map	Classification	ResNet	Expt.: 216 videos	Cross entropy
	Singla et al.^327^	Phase of three wavelengths	Classification	CNN	Expt.: 16,200	---
	Işıl et al.^328^	Phase and amplitude of three wavelengths	Classification	DensNet	Expt.: 33,768	Cross entropy
	Pitkäaho et al.^329^	Phase and amplitude	Classification	CNN	Expt.: ---	---
	Lam et al.^330–332^	Phase and amplitude	Classification	CNN	Sim.: >1000 Expt.: 4000	---
	Terbe et al.^333^	Phase and amplitude in different defocus distances	Classification (7 types)	3D ResNet	Expt.: >9000	Cross entropy
	Wu et al.^334^	Real and imaginary	Classification (5 types)	ResNet	Expt.: 7000	Cross entropy
Imaging modal transformation	Wu et al.^342^	Real and imaginary	Bright-field image	U-Net	Expt.: 30,000 pairs	GAN loss
	Terbe et al.^343^	Amplitude and phase	Bright-field image	U-Net	Expt.: 3000 unpaired	Cycle-GAN loss
	Rivenson et al.^344^	Phase of tissue slices	Stained bright-field image	U-Net	Expt.: >2000 pairs	GAN loss
	Wang et al.^345^	Phase of tissue slices	Stained bright-field or fluorescence image	U-Net	Expt.: 1000 unpaired	Cycle-GAN loss
	Jiang et al.^49^	Amplitude and phase of tissue slices	Stained bright-field or fluorescence image	Y-Net with phase attention	Expt.: ---unpaired	Cycle-GAN loss
	Liu et al.^346^	Amplitude and phase of three wavelength	Stained bright-field image	U-Net	Expt.: 8928 pairs	GAN loss
	Nygate et al.^347^	Phase and gradiences of sperm cells	Stained bright-field image	U-Net	Expt.: 1100 pairs	GAN loss
	Guo et al.^348^	Phase, retardance and Orientation	Fluorescence image	2.5D U-Net	Expt.: 200 full brain sections	*l*_1_-norm
	Kandel et al.^349,350^	Phase	Fluorescence image	U-Net	Expt.: 30–3000 pairs	*l*_2_-norm
	Guo et al.^351^	Phase at different depths	Fluorescence images at different depths	U-Net	Expt.: 200 pairs	*l*_2_-norm
	Chen et al.^352^	Three neighboring phase	Corresponding central fluorescence image	U-Net and EfficientNet	Expt.: 20 z-stacks	*l*_2_-norm
	Jo et al.^353^	3D RI tomogram	3D fluorescence image	3D U-Net	Expt.: 1600 pairs	*l*_2_-norm and gradient difference

“---” indicates not available

### Segmentation

**Fig. 27:**
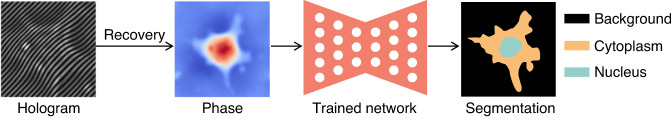
Description of deep-learning-based segmentation from the phase

Image segmentation, aiming to divide all pixels into different regions of interest, is widely used in biomedical analysis and diagnosis. For un-labeled cells or tissues, the contrast of the bright-field intensity is low and thus inefficient to be used for image segmentation. Therefore, segmentation according to the phase distribution of cells or tissues becomes a potentially more efficient way. Given the great success of CNNs in semantic segmentation^280^, it seems that we can easily transplant it for phase segmentation, that is, doing segmentation with the phase as input of the neural network (Fig. 27).

To the best of our knowledge, early in 2013, Yi et al.^281^ first proposed to do segmentation from the phase distribution for the red blood cells, although using a non-learning image-processing-based algorithm. To improve the segmentation accuracy in the case of heavily overlapped and multiple touched cells, they first introduced the fully convolutional network (FCN)^280^ into phase segmentation^282^. Earlier in the same year, Nguyen et al.^283^ used the random forest algorithm to segment prostate cancer tissue from the phase distribution. Ahmadzadeh et al.^284^ used the FCN-based phase segmentation to do nucleus extraction for cardiomyocyte characterization. Subsequently, the U-Net was used for phase segmentation in multiple biomedical applications, such as segmentation of the sperm cells’ ultrastructure for assisted reproductive technologies^285^, SARS-CoV-2 detection^286^, cells live-dead assay^287^, and cells cycle-stage detection^288^. In addition, other types of neural networks were used for phase segmentation, including the mask R-CNN for cancer screening^289^ and the DeepLab-V3+ for cytometric analysis^290^.

Further than the phase, the RI from ODT can be used to segment a sample in three dimensions. Lee et al.^291^ obtained the 3D shape and position of the organelles by 2D segmentation of the RI tomograms at different depths, which are respectively used for the analysis of the morphological and biochemical parameters of breast cancer cells’ nuclei. As a more direct and efficient way, Choi et al.^292^ used a 3D U-Net to segment subcellular compartments directly from a single 3D RI tomogram.

### Classification

Similar but different from the segmentation, the classification task is only responsible for giving the overall category of the input sample image, regardless of the specific pixels in the image. For the classification task, the phase provides more information related to the RI and three-dimensional topography of the sample, making it ideal for transparent samples such as cells, tissues, and microplastics^293,294^. Conventional machine learning algorithms first manually extract tens of features from the phase and then do classification with different models. Support vector machine^295^, as one of the most popular conventional machine learning strategies, is the most used strategy in phase classification^296–303^. In addition, some researchers used other conventional machine learning strategies, such as *k*-nearest neighbor^304,305^, fully-connected neural networks^306,307^, random forest^308,309^, and random subspace^310^. More generally, some researchers compared the accuracy of different conventional machine learning strategies in the same application context^306,311–313^.

**Fig. 28:**
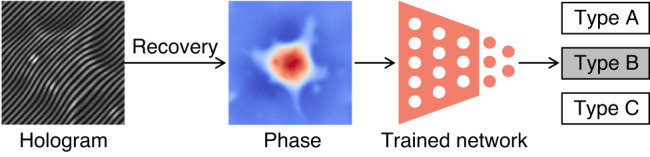
Description of deep-learning-based classification from the phase

Different from conventional machine learning strategies that require manual feature extraction, deep learning usually takes the phase or its further version directly as input, in which the deep CNNs will automatically perform feature extraction (Fig. 28). This automatic feature extraction strategy tends to achieve higher accuracy, but usually requires a larger number of paired input-label datasets as support. The use of phase as input to deep CNNs for classification was first reported in the work of Jo et al.^293^. They revealed that, for cells like anthrax spores, the accuracy of the neural network using phase as input is higher than that of the neural network using binary morphology image obtained by conventional microscopy as input. Subsequently, this deep-learning-based phase classification method has been used in multiple applications, including assessment of T cell activation state^314^, cancer screening^315^, classification of sperm cells under different stress conditions^316^, prediction of living cells mitosis^317^, and classification of different white blood cells^318^. Accuracy in these applications is generally higher than 95% for the binary classification, but cannot achieve comparable accuracy in multi-type classification.

On the one hand, combining the automatically extracted features of the neural network and the manually extracted features for classification can effectively improve the accuracy, which is because the manually extracted features add the prior of human experts to the classifier^319–321^. For instance, after adding the manual morphological features, the accuracy and area under the curve of healthy and sickle red blood cells classification are improved from 95.08% and 0.9665 to 98.36% and 1.0000, respectively^320^. On the other hand, the classification accuracy can also be enhanced by using higher dimensional data of the phase or other data together with the phase as the input of the neural network, such as 3D RI tomogram from the phase^322,323^, more phase in temporal dimension^324–326^, more phase in wavelength dimension^327,328^, and amplitude together with the phase^329–334^.

**Fig. 29:**
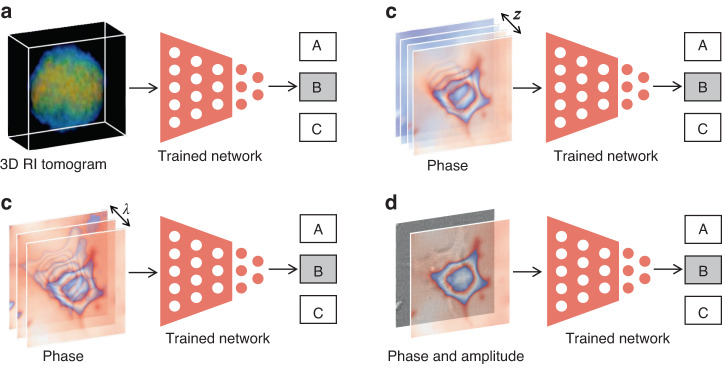
Description of deep-learning-based classification from higher dimensional data of phase. **a** Classification from 3D RI tomogram. **b** Classification from more phase in the temporal dimension. **c** Classification from more phase in wavelength dimension. **d** Classification from amplitude together with the phase. **a** Adapted from ref. ^322^ under Creative Commons (CC BY 4.0) license

#### 3D RI tomogram from the phase (Fig. 29a)

Ryu et al.^322^ used the 3D RI tomogram as the input of a neural network to classify different types of cells, and achieved an accuracy of 99.6% in the binary classification of lymphoid and myeloid cells, and of 96.7% even in five-type classification of white blood cells. For the multi-type classification, they also used the amplitude or phase of the same sample as input to train and test the same neural network, but only achieved an accuracy of 80.1% and 76.6%, respectively. Afterward, Kim et al.^323^ from the same group applied this technology to microbial identification and reached 82.5% accuracy from an individual bacterial cell or cluster for the identification of 19 bacterial species.

#### More phase in temporal dimension (Fig. 29b)

Wang et al.^324^ used the amplitude and phase from time-lapse holograms as inputs to a pseudo-3D CNN to classify the type of growing bacteria, shortening the detection time by >12 h compared with the environmental-protection-agency-approved methods. Likewise, Liu et al.^325^ used the phase from time-lapse holograms as neural network inputs to infer the plaque-forming units probability for each pixel, achieving >90% plaque-forming units detection rate in <20 h. By contrast, Batuch et al.^326^ proposed to use the phase at a specific moment and the corresponding spatiotemporal fluctuation map as the inputs of a neural network to improve the accuracy of cancer cell classification.

#### More phase in wavelength dimension (Fig. 29c)

Singla et al.^327^ used the amplitude and phase of the red-green-blue color wavelengths as inputs of a neural network, thereby achieving a classification accuracy of 97.7% for healthy and malaria-infected red blood cells, and classification accuracy of 91.2% even for different stages of malaria-infection. Similarly, With the blessing of information from the red-green-blue color holograms, Isil et al.^328^ achieved the high-accuracy four-type classification of algae, including accuracy of 94.5%, 96.7%, and 97.6% for D. tertiolecta, Nitzschia, and Thalassiosira algae, respectively.

#### Amplitude together with the phase (Fig. 29d)

Lam et al.^330,331^ used the amplitude and phase as the inputs of a neural network to do the classification of occluded and/or deformable objects, and achieved accuracy over 95%. With the same strategy, they performed a ten-type classification for biological tissues with an accuracy of 99.6%^332^. Further, Terbe et al.^333^ proposed to use a type of volumetric network input by supplementing more amplitude and phase in different defocus distances. They built a more challenging dataset with seven classes by alga in different counts, small particles, and debris. The network with volumetric input outperforms the network with a single amplitude and phase inputs in all cases by ~4% accuracy. Similarly, Wu et al.^334^ used real and imaginary parts of the complex field as network input to do a six-type classification for bioaerosols, and achieved an accuracy of over 94%.

In pursuit of extreme speed for real-time classification, some researchers also choose to directly use the raw hologram recorded by the sensor as the input of the neural network to perform the classification tasks^335–339^. Since the information of amplitude and phase are encoded within a hologram, the hologram-trained neural network should achieve satisfactory accuracy with the support of sufficient feature extraction capabilities, which has been proven in practices including molecular diagnostics^335^, microplastic pollution assessment^336–338^, and neuroblastoma cells classification^339^.

### Imaging modal transformation

**Fig. 30:**
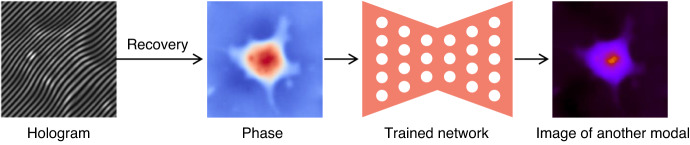
Description of deep-learning-based imaging modal transformation

Let us start this subsection with *image style transfer*^340,341^, which aims to transfer a given image to another specified style under the premise of retaining the content of this image as much as possible. For a type of biological sample or its tissue slice, different parts have different RI properties, different absorption properties, and different chemical or fluorescent staining properties. These four corresponding properties point to phase recovery/imaging, bright-field imaging, and chemical- or fluorescent-staining imaging, respectively, which makes it possible to achieve *image style transfer* from phase recovery to other imaging modals (Fig. 30).

#### From phase recovery to bright-field imaging

**Fig. 31:**
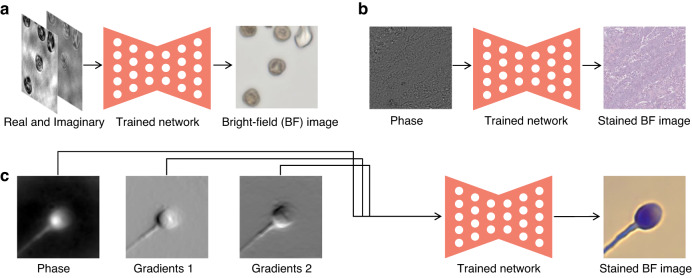
Description of deep-learning-based virtual staining. **a** Inferring bright-field image from real and imaginary parts. **b** Inferring stained bright-field image from the phase. **c** Inferring stained bright-field image from the phase and its gradients. **a**, **b** Adapted from refs. ^342,344^ under Creative Commons (CC BY 4.0) license. **c** Adapted from ref. ^347^ under Creative Commons (CC BY-NC-ND 4.0) license

The bright-field images of some color biological samples have sufficient contrast due to their strong absorption of visible light, so for such samples, bright-field imaging can be used as the target imaging modality, in which a neural network is used to transfer the complex-value image of the sample into its virtual bright-field image. In 2019, Wu et al.^342^ presented the first implementation of this idea, called bright-field holography, in which a neural network was trained to transfer the back-propagated complex-value images from a single hologram to their corresponding speckle- and artifact-free bright-field images (Fig. 31a). This type of “bright-field holography” is able to infer a whole 3D volumetric image of a color sample like pollen from its single-snapshot hologram. Further, Terbe et al.^343^ implemented “bright-field holography” with a cycle-GAN in the case of unpaired datasets.

#### From phase recovery to chemical-staining imaging

For most transparent/colorless biological samples, chemical staining enables them to be clearly observed or imaged under bright-field microscopy. This allows the above “bright-field holography” to be used for transparent biological samples as well, which is called virtual staining. It directly infers the corresponding digital stained image from the phase recovered by label-free methods, which avoids the complicated, time-consuming, and contaminating staining processes. Rivenson et al.^344^ applied this virtual staining technique to the inspection of histologically stained tissue slices and named it PhaseStain, in which a well-trained neural network was used to directly transfer the phase of tissue slices to their bright-field image of virtual staining (Fig. 31b). Using label-free slices of human skin, kidney, and liver tissue, they conducted an experimental demonstration of the efficacy of “PhaseStain” by imaging them with a holographic microscope. The resulting images were compared to those obtained through bright-field microscopy of the same tissue slices that were stained with HandE, Jones’ stain, and Masson’s trichrome stain, respectively. The reported “PhaseStain” greatly saves time and costs associated with the staining process. Similarly, Wang et al.^345^ applied the “PhaseStain” in Fourier ptychographic microscopy and adapted it to an unpaired dataset with a cycle-GAN. Further, by introducing the phase attention guidance, Jiang et al.^49^ addressed the ambiguity problem of intensity- or phase-only networks for virtual staining. Liu et al.^346^ used six images of amplitude and phase at three wavelengths as network input to infer the corresponding virtual staining version. In addition to tissue slices, Nygate et al.^347^ demonstrated the advantages and potential of this deep learning virtual staining approach on a single biological cell like sperm (Fig. 31c). To improve the effectiveness of virtual staining, they used the phase gradients as an additional hand-engineered feature along with the phase as the input of the neural network. In order to assess the effectiveness of virtual staining, they used virtual staining images, phase, phase gradients, and stain-free bright-field images as input data for the five-type classification of sperm, and found that the recall values and F1 scores of virtual staining images were higher than those of other data twice or even four times. This type of single-cell staining approach provides ideal conditions for real-time analysis, such as rapid stain-free imaging flow cytometry.

#### From phase recovery to fluorescent-staining imaging

**Fig. 32:**
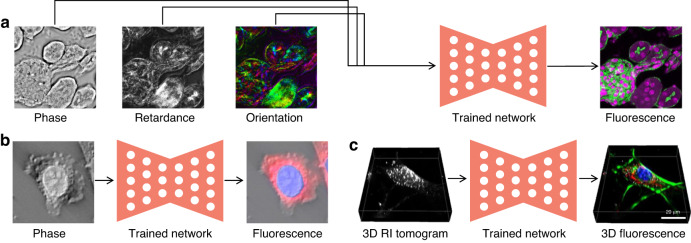
Description of deep-learning-based label-free virtual fluorescence imaging. **a** Inferring fluorescence image from the phase, retardance, and orientation. **b** Inferring fluorescence image from the phase. **c** Inferring 3D fluorescence image from a 3D RI tomogram. **a**, **b** Adapted from refs. ^348,349^ under Creative Commons (CC BY 4.0) license. **c** Adapted from ref. ^353^ with permission of Springer Nature

Apart from imaging color or chemical-stained biological samples with bright-field microscopy, fluorescence microscopy can provide molecular-specific information by imaging fluorescence-labeled biological samples. As a labeled imaging method, fluorescence microscopy has insurmountable disadvantages, including phototoxicity and photobleaching. Guo et al.^348^ proposed the concept of “transferring the physical-specific information to the molecular-specific information via a trained neural network” (Fig. 32a). Specifically, they used the phase and polarization of cell samples as multi-channel inputs to infer the corresponding fluorescence image, and further demonstrated its performance by imaging the architecture of brain tissue and prediction myelination in slices of a developing human brain. Almost simultaneously, Kandel et al.^349^ used a neural network to infer the fluorescence-related subcellular specificity from a single phase, which they called phase imaging with computational specificity (Fig. 32b). With these label-free methods, they monitored the growth of both nuclei and cytoplasm for live cells and the arborization process in neural cultures over many days without loss of viability^350^. Guo et al.^351^ further inferred the fluorescence images from the phase at different depths and performed 3D prediction for mitochondria. The above methods are performed on wide-field fluorescence microscopes, which cannot provide high-resolution 3D fluorescence data for neural networks as ground truth. Hence, Chen et al.^352^ presented an artificial confocal microscopy consisting of a commercial confocal microscope augmented by a laser scanning gradient light interference microscopy system. It can provide the phase of the samples in the same field of view as the fluorescence channel to obtain paired datasets. With the support of deep learning, their proposed artificial confocal microscopy combines the benefits of non-destructive phase imaging with the depth sectioning and chemical specificity of confocal fluorescence microscopy.

The aforementioned imaging modal transformation methods use phase as the input of neural networks, but the phase, in addition to being related to RI, also depends on the thickness of the biological sample or its tissue slice. Therefore, a neural network trained on the dataset of a biological type is difficult to generalize to another different one. Unlike inferring the fluorescence image from the phase, RI is an absolute and unbiased quantity of biological samples, so a neural network trained with RI as input is naturally applicable to new species. Jo et al.^353^ thus built a bridge from ODT to fluorescence imaging via deep learning (Fig. 32c). They trained a neural network with the 3D RI tomogram as input and the corresponding fluorescence image as ground truth. With the trained neural network, they performed various applications within the endogenous subcellular structures and dynamics profiling of intact living cells at unprecedented scales.

## Conclusion and outlook

The introduction of deep learning provides a data-driven approach to various stages of phase recovery. Based on where they are used, we provided a comprehensive review of how neural networks work in phase recovery. Deep learning can provide pre-processing for phase recovery before it is performed, can be directly used to perform phase recovery, can post-process the initial phase obtained after phase recovery, or can use the recovered phase as input to implement specific applications. Despite the fact that deep learning provides unprecedented efficiency and convenience for phase recovery, there are some common general points to keep in mind when using this learn-based tool.

### Datasets

For the supervised learning mode, a paired dataset provides enough rich and high-quality prior knowledge as a guide for neural network training. As one of the most common ways, some researchers choose to collect the intensity image of the real sample through the experimental setup as the input, and calculate the corresponding phase through conventional model-based methods as ground truth (label). Numerical simulations can be a convenient and efficient way to generate datasets for some cases, such as phase unwrapping^66^, hologram resolution enhancement^74^ and diffractive imaging^130^. The paired dataset thus implicitly contains the input-to-label mapping relationship in a large number of specific samples, which determines the upper limit of the ability of the trained neural network. For instance, if the dataset is collected under fixed settings, the trained neural network can only target a fixed device parameter (such as defocus distance, off-axis angle, and wavelength) or a certain class of samples, but cannot adapt to other situations that are not implied in the dataset. Of course, one can ameliorate this by using different settings and different types of samples when collecting datasets, thereby including various cases in the paired training samples, such as adapting to a certain range of defocus distance^114,166^, adapting to different aberrations^119,129^, adapting to different off-axis angles^123^ and adapting to more types of samples^127^. One can use Shannon entropy to quantitatively represent the richness of the amount of information contained in the dataset, which directly affects the generalization ability of the trained neural network^117^. In addition, the spatial frequency content of the training samples in datasets also limits the ability of the trained neural network to resolve fine spatial features, which can be improved to some extent by pre-processing the power spectral density of the training samples^115^. For the weak-supervised learning mode, the cycle-GAN-based method trains neural networks with an unpaired dataset for learning the mapping relationship between the input domain and the target domain, including phase recovery^124,125,142^, noise reduction^209^, resolution enhancement^227^, and imaging modal transformation^343,345^. As for the unsupervised learning mode, under the guidance of forward physical models and input-only datasets, neural networks learn the inverse process^152,153,159–162^.

### Networks and loss functions

Guided/Driven by the dataset, the neural network is trained to learn the mapping relationship from the input domain to the target domain by minimizing the difference between the actual output and ground truth (loss functions). Therefore, the fitting ability of the neural network itself and the perception ability of the loss function determines whether the implicit mapping relationship in the dataset can be well internalized into the neural network. Conventional encoder-decoder-based neural networks have sufficient receptive fields and strong fitting capabilities, but down-sampling operations such as max-pooling lose some high-frequency information. Dilated convolutions can improve the receptive field while retaining more high-frequency information^141^. Convolution in the Fourier frequency domain guarantees a global receptive field, since each pixel in the frequency domain contains contributions from all pixels in the spatial domain^145,146^. In order to make the neural network more focused on different spatial frequency information, one can also use two neural networks to learn the high- and low-frequency bands, respectively, and then use the third neural network to merge them into a full spatial frequency version^169^. Neural architecture search is another potential technology that automatically searches out the optimal network structure from a large structure space^147^. In addition to the aforementioned CNNs, due to the excellent global feature perception, Vision Transformer^112^ and Swin Transformer^143^ achieved better inference performance than classic CNNs in autofocusing^108^ and phase recovery^142^. However, it should be noted that Transformer does not have inherent translational equivariance and invariance like CNNs, and thus requires corresponding data enhancement. The recently proposed local conditional neural fields framework is expected to achieve highly generalized multi-scale phase recovery, in which generalization ability comes from measurement-specific information in latent space while multi-scale ability comes from local representation^354^. As the most commonly used loss functions, *l*_2_-norm and *l*_1_-norm are more responsive to low-frequency information and less sensitive to high-frequency information. That is to say, the low-frequency information in the output of the neural network contributes more to the *l*_2_-norm and *l*_1_-norm loss functions than the high-frequency information. Therefore, some researchers have been trying to find more efficient loss functions, such as NPCC^115^, GAN loss^132,139,140^, and default feature perceptual loss of VGG layer^168^. So far, what kind of neural network and loss function is the best choice for phase recovery is still inconclusive.

### Network-only or physics-connect-network (PcN)

Network-only strategy aims to infer the final phase from the raw measured intensity image in an end-to-end fashion using a neural network. It’s a one-shot approach, letting the neural network do it all in one go. Neural networks not only need to perform regularization to remove twin-image and self-interference-related spatial artifacts but also undertake the task of free-space light propagation. Therefore, the inference results of the network-only strategy are not satisfactory in some severely ill-posed cases, including weak-light illumination^118^ and dense samples^137^. Since free-space light propagation is a well-characterized physical model that can be reproduced and enforced numerically, using numerical propagation in front can relieve the burden on the neural network and allow it to focus on learning regularization. In fact, PcN can indeed infer better results than network-only in the above ill-posed cases^118,137^. In another similar scheme, the neural network only performs the task of hologram generation before the phase-shifting algorithm, thus achieving better generalization ability than network-only^89^. In addition, using speckle-correlation processing before the neural network makes the trained neural network suitable for unknown scattering media and target objects^355^.

### Interpretability

In phase recovery, learning-based deep learning techniques usually attempt to automatically learn a specific mapping relationship by optimizing/training neural network parameters with the real-world paired dataset. Deep neural networks usually adopt a multi-layer architecture and contain a large number of trainable parameters (even greater than millions), and are thus capable of learning complicated mapping relationships from datasets. Unlike physics-based algorithms, such network architectures that are general to various tasks often lack interpretability, meaning that it is difficult to discover what the neural network has learned internally and what the role of a particular parameter is by examining the trained parameters. This makes one helpless in practical applications when encountering a failure of neural network inference, in which they can neither analyze why the neural network failed for that sample nor make targeted improvements for the neural network to avoid this failure in subsequent uses. The algorithm unrolling/unfolding technique proposed by Gregor and LeCun gives hope for the interpretability of neural networks^199^, in which each iteration of physics-based iterative algorithms is represented as one layer of the neural network. One inference through such a neural network is equivalent to performing a fixed number of iterations of the physics-based iterative algorithm. Usually, physics-based parameters and regularization coefficients are transferred into the unrolled network as trainable parameters. In this way, the trained unrolled network can be interpreted as a physics-based iterative algorithm with a fixed number of iterations. In addition, the unrolled network naturally inherits prior structures and domain knowledge from a physics-based iterative algorithm, and thus its parameters can be efficiently trained with a small dataset.

### Uncertainty

When actually using a trained neural network to do inference for a tested sample, its ground truth is usually unknown, which makes it impossible to determine the reliability of the inferred results. To address this, Bayesian CNNs perform phase inference while giving uncertainty maps to describe the confidence measure of each pixel of the inferred result^132,356–358^. This uncertainty comes from both the model itself and the data, called epistemic uncertainty and aleatoric uncertainty, respectively. The network-output uncertainty maps are experimentally verified to be highly consistent with the real error map, which makes it possible to assess the reliability of inferred results in practical applications without any ground truth^132,358^. In addition to Bayesian neural networks, there are three other uncertainty estimation techniques, including single deterministic methods, ensemble methods, and test time augmentation methods^359^.

### From electronic neural networks to optical neural networks

So far, the artificial neural networks involved in this review mostly run in the hardware architecture with electronics as the physical carrier, such as the graphic processing unit, which is approaching its physical limit. Replacing electrons with photons is a potential route to high-speed, parallel, and low-power artificial intelligence computing, especially optical neural networks^360,361^. Among them, spatial-structure-based optical neural networks, represented by the diffractive deep neural network^362^, are particularly suitable for image processing and computational imaging^363–365^. Some examples have initially demonstrated the potential of using optical neural networks for phase recovery^366–368^.

### Inherent limitations of the hardware imaging system

In addition to considering how to use neural networks to better recover phases from measured intensity maps, the capabilities of the hardware imaging system itself to detect and capture information are also essential. This is because a clear understanding exists that even the most advanced deep learning techniques cannot recover information that the hardware imaging systems have not recorded. In the case of lensless systems, incorporating additional light field modulation devices, such as coded layers, can transform otherwise imperceptible low- and high-frequency information into detectable levels^49–52^. A potential research direction involves using deep learning to design coded layer distributions that optimally consider information across all frequencies. For lens-based systems, the illumination strategy dictates the frequency content entering the effective numerical aperture. Hardware parameters, such as illumination patterns, can be integrated as trainable parameters within the PiN-based phase-recovery neural network, allowing for joint optimization through training datasets^369,370^.

Learning-based deep neural networks have enormous potential and efficiency, while conventional physics-based methods are more reliable. We thus encourage the incorporation of physical models with deep neural networks, especially for those well modeling from the real world, rather than letting the deep neural network perform all tasks as *a black box*. A possible way is to thoroughly consider the network structure, loss function, and priors from both the dataset and physical model during the training stage to obtain an effective pre-trained neural network; in actual use, the pre-trained neural network can be employed for one-time inference to address situations requiring high real-time requirements, or alternatively, the physical model can be used to iteratively fine-tune the pre-trained neural network to achieve higher accuracy.
